# Clustering Patients with Pulmonary Hypertension Using the Plasma Proteome

**DOI:** 10.1164/rccm.202408-1574OC

**Published:** 2025-05-09

**Authors:** Athénaïs Boucly, Shanshan Song, Merve Keles, Dennis Wang, Luke S. Howard, Marc Humbert, Olivier Sitbon, Allan Lawrie, A. A. Roger Thompson, Philipp Frank, Mika Kivimaki, Christopher J. Rhodes, Martin R. Wilkins

**Affiliations:** ^1^ National Heart and Lung Institute, Faculty of Medicine, Imperial College London, London, United Kingdom;; ^2^ Université Paris-Saclay, INSERM UMR_S 999 (HPPIT), Service de Pneumologie et Soins Intensifs Respiratoires, Hôpital Bicêtre, AP-HP, Le Kremlin-Bicêtre, France;; ^3^ Bioinformatics Institute, Agency for Science, Technology, and Research (A*STAR), Singapore, Republic of Singapore;; ^4^ National Pulmonary Hypertension Service, Imperial College Healthcare NHS Trust, Hammersmith Hospital, London, United Kingdom;; ^5^ Division of Clinical Medicine, The University of Sheffield, Sheffield, United Kingdom;; ^6^ Brain Sciences, University College London, London, United Kingdom; and; ^7^ Clinicum, University of Helsinki, Helsinki, Finland

**Keywords:** classification, proteomics, pulmonary hypertension, theragnostics

## Abstract

**Background:**

Patients with pulmonary hypertension are classified according to clinical criteria to inform treatment decisions. Knowledge of the molecular drivers of pulmonary hypertension might better inform treatment choice.

**Objectives:**

To investigate plasma protein clusters in patients with a diagnosis of pulmonary hypertension.

**Methods:**

Between 2013 and 2021, 470 patients with pulmonary hypertension, 136 control subjects with symptomatic disease, and 59 healthy control subjects were enrolled as a discovery cohort. Plasma levels of 7,288 proteins were assayed (SomaScan 7K platform). Proteins that distinguished pulmonary hypertension from both control groups were selected for unsupervised clustering (k-means clustering of Uniform Manifold Approximation and Projection dimensions). Clinical characteristics and outcomes were compared across clusters. Separate cohorts of serially sampled patients from pulmonary hypertension centers in the United Kingdom (*n* = 229) and France (*n* = 79) provided independent validation.

**Measurements and Main Results:**

A total of 156 plasma proteins that distinguished pulmonary hypertension from control subjects with symptomatic disease and healthy control subjects formed four clusters with diverse 5-year survival rates: 78% (cluster 4), 62% (cluster 2), 44% (cluster 3), and 33% (cluster 1). The distinction and clinical relevance of the clusters were confirmed in validation cohorts by their association with survival. To further characterize the therapeutic relevance of the clusters, we investigated two experimental drug targets: the PDGF (Platelet-Derived Growth Factor) pathway was upregulated in cluster 3 compared with other clusters, and the TGF-β (Transforming Growth Factor-β) pathway was upregulated in cluster 1.

**Conclusions:**

Plasma proteomic profiling of patients with pulmonary hypertension distinguishes four clusters, independent of the clinical classification. These groups, based on differential plasma protein levels, could act as theragnostic biomarkers for new therapies targeting PDGF and TGF-β pathways.

**At a Glance Commentary**

**Scientific Knowledge on the Subject**

It is recognized that the plasma proteome (by acting as a “liquid biopsy”) has the potential to provide a deep molecular phenotype in pulmonary hypertension and enable personalized medicine. Studies to date have been largely confined to patients with pulmonary arterial hypertension and focused on prognostic markers for risk assessment, rather than their use as theragnostics.

**What This Study Adds to the Field**

Through unsupervised clustering of the plasma proteome in a broad population of patients with clinically defined pulmonary hypertension, this study identified four patient groups linked to underlying molecular pathways, independent of the current clinical classification. The differential expression of PDGF and TGF-β pathways across the proteome clusters offers the opportunity for plasma proteomic profiling to select patients for studies of drugs targeting these pathways. The findings lay the foundation for the precise targeting of patients with tailored therapeutics according to molecular data.

Pulmonary hypertension (PH) can present in relative isolation or as a comorbidity in left heart failure, chronic lung disease, and other conditions (1, 2). It causes death from right heart failure and remains a formidable challenge for therapeutic drug development (1, 2). The first step in management is the classification of a patient into one of five clinical groups, which guides treatment strategy (1, 2). Classification into a single group can be problematic, as up to 40% of patients show mixed etiology (3). Moreover, relying on clinical characteristics and measurements does little to define critical drug targets and aid new drug development.

Proteomics is a powerful tool for unraveling the intricate molecular landscape of diseases (4). The plasma proteome comprises several thousand circulating proteins secreted or leaked from tissues (5, 6). To date, the focus of high-throughput plasma proteomics in PH has been to identify key circulating markers of disease progression or treatment response in group 1 patients with pulmonary arterial hypertension (PAH; precapillary PH that may be idiopathic, heritable, or associated with drug exposure, connective tissue disease, and congenital heart disease) (7–10). However, this focus on group 1, and the assignment on clinical criteria of some patients with PAH to other PH groups, particularly group 2 (left heart failure) and group 3 (lung disease) (3), may undermine the insights the plasma proteome can provide into finding new drug targets and therapeutic options. We argue that in-depth molecular profiling applied to the broader population of patients with a clinical diagnosis of PH is a better approach to developing targeted treatments for PH (11).

Here we use unsupervised clustering of plasma proteins from patients with clinically defined PH to identify robust protein signatures independent of the clinical classification, with the overarching goal of paving the way for more personalized and targeted therapeutic strategies.

## Methods

### Discovery Cohort

The discovery study population comprised patients with suspected PH who attended Imperial College NHS Trust between 2013 and 2021. Patients with PH were classified in group 1 (PAH), group 2 (PH associated with left heart disease, PH-LHD), group 3 (PH associated with lung disease, PH-lung), or group 4 (chronic thromboembolic PH, CTEPH), using European Society of Cardiology/European Respiratory Society guidelines (12, 13). Patients referred with suspected PH but with a mean pulmonary artery pressure (mPAP) <25 mm Hg on right heart catheterization were classified as NoPH (symptomatic disease) control subjects. Contemporaneous plasma samples were obtained from volunteers without cardiovascular or respiratory diseases (healthy control subjects). All patients were recruited with informed written consent and local research ethics committee approval (11/LO/0395 and 17/LO/0563). Sample collection and processing are detailed in the supplemental methods.

### Validation Cohorts

Separate cohorts of patients with PH with serial plasma samples collected over the same time period were used for independent validation: the UK National Cohort Study (NCT01907295), the French EFORT study (Evaluation of Prognostic Factors and Therapeutic Targets in PAH; NCT01185730), and the Sheffield Teaching Hospitals Observational Study of patients with PH, Cardiovascular or Respiratory Disease (18/YH/0441). The Whitehall II study (14) provided a dataset on samples collected from a large cohort that were healthy at baseline.

### Selecting Relevant Proteins

Patients from the discovery cohort were randomized into training (80%) and replication groups (20%). Protein levels were compared between patients with PH and both healthy and NoPH control subjects by logistic regression models, correcting for age, sex, and principal component outliers (*see* Figure E1 in the online supplement). All comparisons were corrected for multiple testing using Benjamini-Hochberg false discovery rate. A threshold of *q* < 0.05 was considered statistically significant.

To identify the combination of proteins that best predicted PH diagnosis, a least absolute shrinkage and selection operator (LASSO) modeling approach was applied (9), with the regularization parameter determined by the lowest error plus 1 SE using the *glmnet* R-package (15). Similar analyses were performed for proteins that distinguished patients with PH and control subjects to identify the combination of proteins that best reflected PH pathology. Performance of these models was tested in the replication group by receiver operating characteristic analyses using the *pROC* R-package.

### Clustering of Patients with PH Using Proteins

Proteins that distinguished patients with PH from both healthy control subjects and NoPH control subjects (in models corrected for age, sex, principal component outliers, hemolysis, coagulation Factor X, and cystatin C) were taken forward for dimensional reduction using the Uniform Manifold Approximation and Projection (UMAP) R-package, followed by cluster analysis of protein-derived UMAP dimensions using the *NbClust* R-package. Demographic and clinical differences between the clusters were assessed by nonpaired ANOVA, Kruskal-Wallis, and chi-square tests. We compared survival in the different clusters by log-rank test, from plasma sampling to death or censoring. We trained a random forest model to classify new samples for cluster membership to validate our findings in independent cohorts. The classifier can be downloaded from https://doi.org/10.5281/zenodo.14509735.

### Pathway Enrichment

Molecular enrichment analysis was performed using the *WebGestaltR* R-package. Heatmaps of proteins within pathways of PAH drugs in development were performed using *gplots* and *pheatmap* R-packages. The relative fluorescence of proteins of interest in the clusters were compared by nonpaired ANOVA tests with Dunnett’s multiple pairwise comparisons.

**Figure 1.**
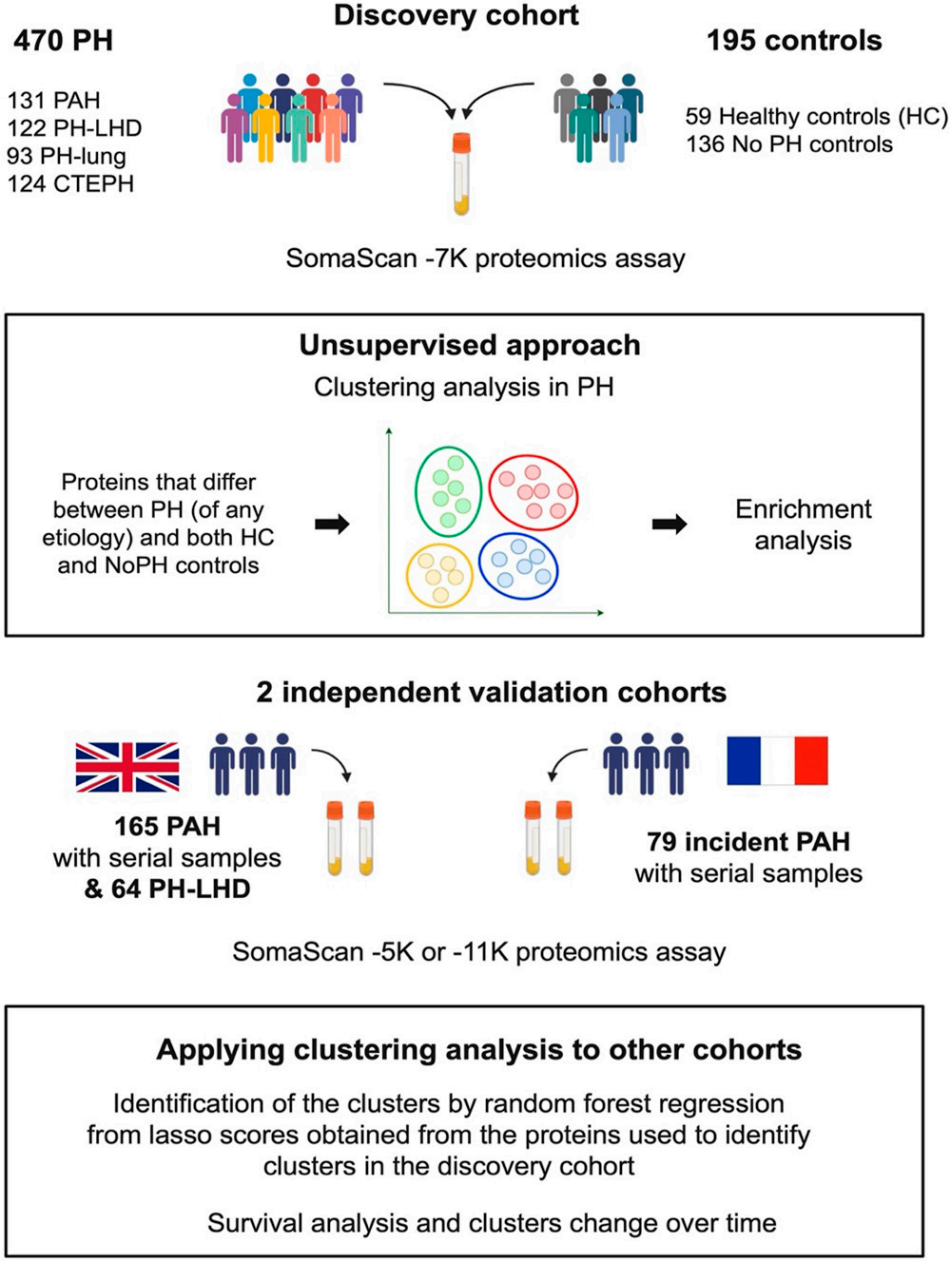
Overview of study design. Circulating levels of 7,288 proteins were assayed (SomaScan 7K platform) in plasma samples from 470 patients with PH, irrespective of clinical pulmonary hypertension subgroup; 136 subjects with symptomatic disease; and 59 healthy control subjects enrolled as a discovery cohort. Proteins that distinguished pulmonary hypertension from both control groups were selected for unsupervised clustering (k-means clustering of Uniform Manifold Approximation and Projection dimensions). Separate cohorts of serially sampled patients from the UK (*n* = 229) and France (*n* = 79) provided independent validation of the clusters and dynamic association with clinical status. Enrichment analysis was used to identify key molecular pathways in each cluster. CTEPH = chronic thromboembolic PH; HC = healthy control subjects; No PH = subjects with symptomatic disease without PH; PAH = pulmonary arterial hypertension; PH = pulmonary hypertension; PH-LHD = PH associated with left heart disease; PH-lung = PH associated with lung disease.

Statistical analysis was performed in R (version 4.3.1) and SPSS (version 29; IBM). An overview of the methodology is displayed in Figure 1 and in the supplemental methods.

## Results

### Study Populations

Our discovery study population comprised 470 patients with PH, classified as PAH (*n* = 131), PH-LHD (*n* = 122), PH-lung (*n* = 93), and CTEPH (*n* = 124), together with 136 NoPH (control subjects with symptomatic disease) and 59 healthy control subjects (Figure 1 and Tables 1 and E1). Among patients with PH, 379 (81%) were newly diagnosed with PH (i.e., incident patients). The mean age was 64 ± 16 years; 56% were female, and 74% were in functional class III. All individuals were randomized into training (80%) and replication (20%) subgroups (Table E2) for initial analysis. Validation was conducted on two independent PAH cohorts: one prevalent PAH cohort from the United Kingdom (*n* = 165 patients, including 125 with serial samples) and one incident PAH cohort from France with serial samples (*n* = 79), and a separate UK PH-LHD group (*n* = 64; Table E3). The Whitehall II study (14) (Table E4) provided an independent healthy control population (*n* = 6,196) as a negative control.

**Table 1.** Demographics and Clinical and Hemodynamic Characteristics of the Study Population

	Healthy Control Subjects (*n* = 59)	No PH Control Subjects (*n* = 136)	Pulmonary Hypertension (*n* = 470)
Sex female/male	41 (69)/18 (31)	87 (64)/49 (36)	262 (56)/208 (44)
Age, yr	46 ± 12	61 ± 16	64 ± 16
Race			
White	38 (64)	92 (68)	348 (74)
African	2 (3)	15 (11)	28 (6)
Asian	8 (14)	8 (6)	30 (6)
No data	11 (19)	21 (15)	64 (14)
Treatment-naive patients	59 (100)	136 (100)	379 (81)
Systemic hypertension	0	70 (51)	147 (31)
Diabetes mellitus	0	16 (12)	68 (14)
Ischemic heart disease	0	6 (4)	22 (5)
Atrial fibrillation permanent	0	15 (11)	68 (14)
Thyroid disease	0	1 (1)	18 (4)
COPD	0	9 (7)	45 (10)
No comorbidity	59 (100)	34 (25)	139 (30)
Sampled within 7 days of first diagnosis/treatment	NA	116 (85)	309 (66)
NYHA FC I–II/III/IV	NA	47 (35)/85 (63)/3 (2)	80 (17)/347 (74)/41 (9)
6MWD, m	NA	312 ± 139	240 ± 147
BNP, ng/L	NA	48 (16–141)	166 (57–440)
RAP, mm Hg	NA	8 ± 4	10 ± 5
mPAP, mm Hg	NA	22 ± 9	43 ± 10
PAWP, mm Hg	NA	12 ± 4	12 ± 6
Cardiac output, L/min	NA	6.4 ± 2.7	4.4 ± 1.8
Cardiac index, L/min/m^2^	NA	3.3 ± 1.5	2.4 ± 0.9
PVR, WU	NA	1.6 ± 0.9	8 ± 5
S$\bar{v}$_O_2__, %	NA	76 ± 7	74 ± 13

*Definition of abbreviations*: 6MWD = 6-minute-walk distance; BNP = brain natriuretic peptide; COPD = chronic obstructive pulmonary disease; mPAP = mean pulmonary arterial pressure; NA = not applicable; NYHA FC = New York Heart Association functional class; PAWP = pulmonary arterial wedge pressure; PVR = pulmonary vascular resistance; RAP = right atrial pressure; S$\bar{v}$_O_2__ = mixed venous oxygen saturation; WU = Wood units.

Data are presented as *n* (%), mean ± SD, or median (interquartile range).

### Plasma Proteome Profiles

Principal component analysis was performed to evaluate variation in protein expression profiles and identify patterns across the samples. The percentage of variance explained by each principal component is provided in Table E5. Using standard supervised analysis comparing patients with PH and control subjects (detailed in the online supplement), plasma proteins that differed by circulating level between PH and both healthy and NoPH control subjects were used to construct models among patients with PH to distinguish the main clinical PH groups (Tables E6–E8 and Figures E1–E6). There was significant overlap in the proteins associated with each PH subgroup (all pairs *P* < 0.001, Fisher’s exact test; Figure E1), suggesting important molecular clusters across these clinical groups.

### Unsupervised Cluster Analysis of All Proteomes of Patients with PH

**Figure 2.**
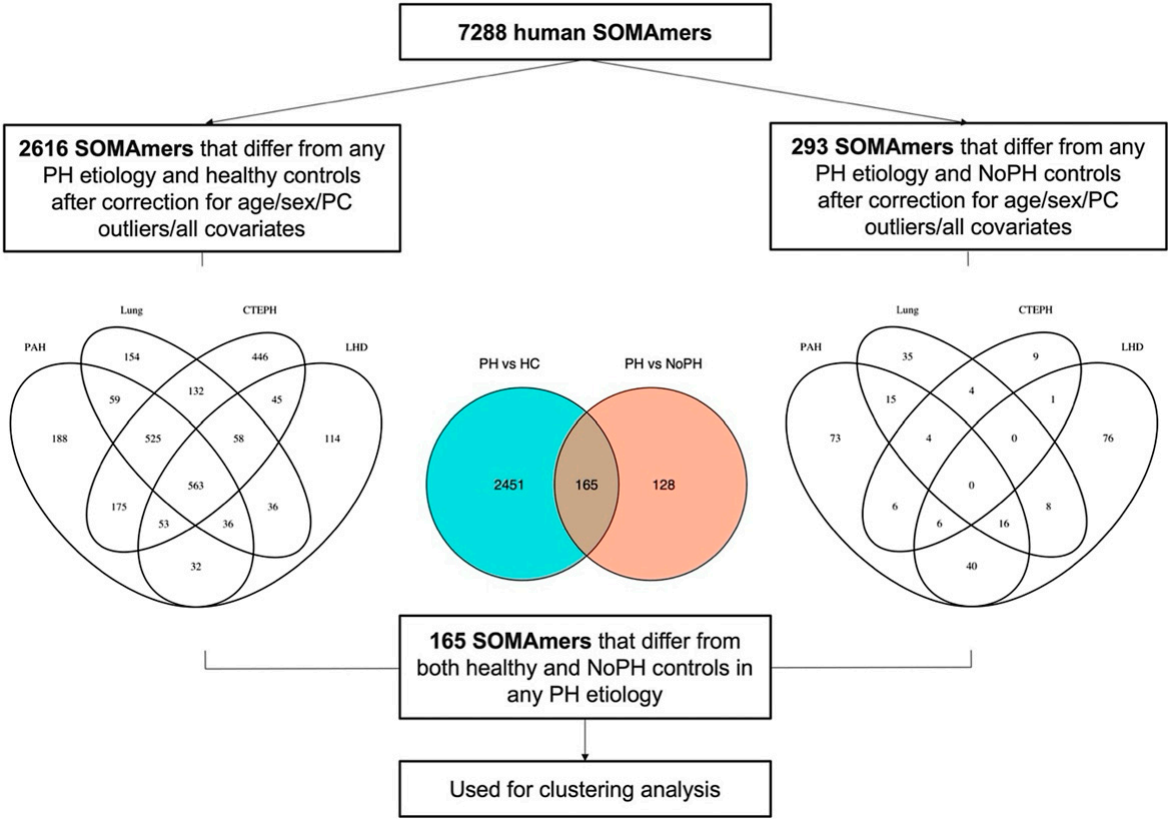
Methodology used to select SOMAmers used for clustering analysis. Proteins that distinguished patients with PH (in at least one etiological diagnostic group) from both healthy control subjects and NoPH control subjects (in models corrected for age, sex, principal component outliers, hemolysis, coagulation Factor X, and cystatin C) were used for clustering analysis. Venn diagrams indicate the overlap of proteins identified in each analysis run and the final selection of 165 SOMAmers measuring 156 unique proteins. HC = healthy control subjects; NoPH = subjects with symptomatic disease without PH; PC = principal components (of protein levels); PH = pulmonary hypertension.

**Figure 3.**
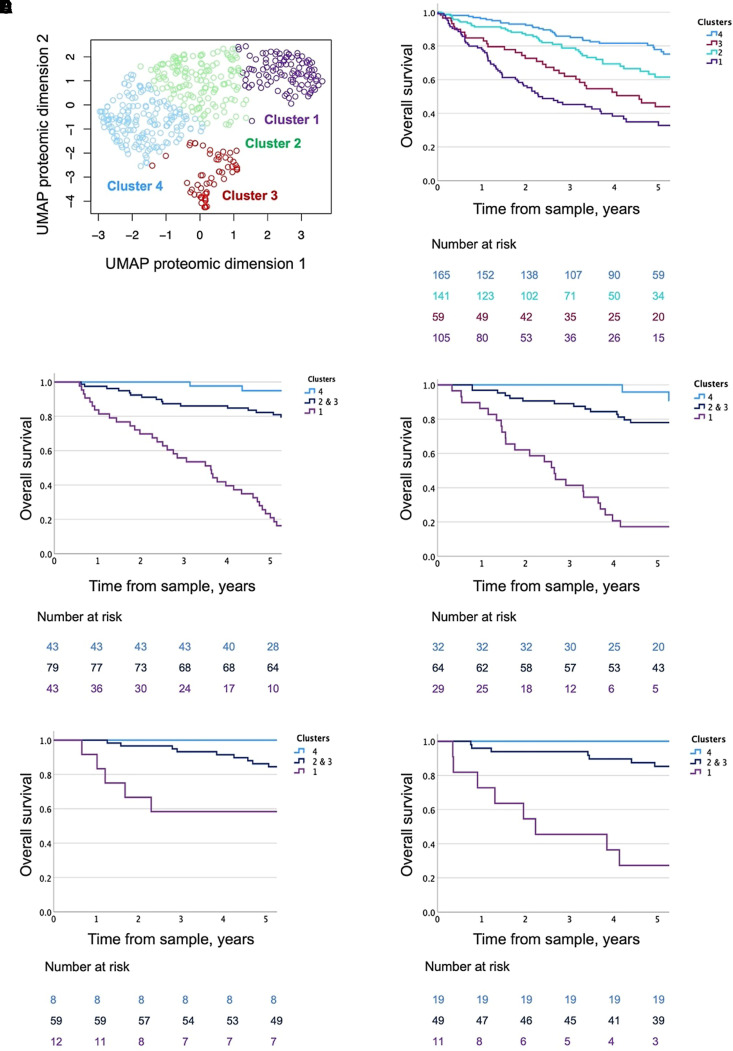
Proteomic clusters. (*A*) UMAP of the 156 proteins used for clustering analysis, and (*B*) Kaplan-Meier survival curves according to clusters in the discovery cohort and (*C* and *D*) UK baseline (*C*) and first follow-up visits (*D*), and (*E* and *F*) French pulmonary arterial hypertension baseline (*E*) and first follow-up visits (*F*) validation cohorts. (*A*) Each color corresponds to a cluster identified by k-means clustering analysis. (*B*) Survival curves of patients classified in cluster 1 (purple), 2 (green), 3 (red), 4 (blue). Log rank test, *P* < 0.001. *C–F*: survival curves of patients classified in cluster 1 (purple), 2 or 3 (dark blue), 4 (blue). Log rank test, *P* < 0.001 for each analysis (*C–F*). UMAP = Uniform Manifold Approximation and Projection.

To identify novel proteomic clusters, we focused on proteins associated with PH irrespective of clinical group and robust to potential confounders. Plasma levels of 165 SOMAmers (targeting 156 proteins) differed significantly between PH (of any etiology) and both NoPH and healthy control subjects after correction for age, sex, principal component outliers, hemolysis, coagulation Factor X, and cystatin C (Figure 2). The dimensions of this dataset of proteins were reduced by UMAP. Unsupervised k-means clustering analysis of the proteomic UMAP dimensions of all 470 patients with PH revealed that, with a substantial stability rate of 89%, the optimal number of clusters was four (Figure 3A), supporting a robust and consistent clustering of patients, which was visually apparent (Figure E7).

Patients in cluster 4 were younger and had fewer comorbidities than the others, whereas patients in cluster 1 had more severe PH (Table 2). After a median of 3.2 years (interquartile range, 1.8–5.3 yr) from plasma sampling, 188 (40%) patients had died. Events occurred in 65% of cluster 1, 59% of cluster 3, 33% of cluster 2, and 23% of cluster 4. At 5 years, the Kaplan-Meier survival rate was divergent (log rank test, *P* < 0.001; Figure 3B): highest in cluster 4 (78%), lowest in cluster 1 (33%), and intermediate for clusters 2 (62%) and 3 (44%).

**Table 2.** Demographic, Functional, Exercise, and Hemodynamic Characteristics of Pulmonary Hypertension According to Clusters

	Cluster 1 (*n* = 105)	Cluster 2 (*n* = 141)	Cluster 3 (*n* = 59)	Cluster 4 (*n* = 165)	*P* Value
Sex, female/male	45 (43)/60 (57)	81 (57)/60 (43)	35 (59)/24 (41)	101 (61)/64 (39)	0.023
Age, yr	69 ± 12	64 ± 16	67 ± 16	59 ± 16	<0.001
Race					
White	83 (79)	97 (69)	45 (76)	123 (75)	0.18
African	1 (1)	11 (8)	2 (3.5)	14 (8)	
Asian	7 (7)	13 (9)	2 (3.5)	8 (5)	
No data	14 (13)	20 (14)	10 (17)	20 (12)	
PH etiology					<0.001
Pulmonary arterial hypertension	24 (23)	48 (34)	12 (20)	47 (28)	
PH associated with LHD	38 (36)	43 (31)	14 (24)	27 (16)	
PH associated with lung disease	26 (25)	16 (11)	17 (29)	34 (21)	
CTEPH	17 (16)	34 (24)	16 (27)	57 (35)	
Systemic hypertension	32 (30)	57 (40)	17 (29)	41 (25)	0.031
Diabetes mellitus	16 (15)	22 (16)	7 (12)	23 (14)	0.906
Ischemic heart disease	7 (7)	7 (5)	3 (5)	5 (3)	0.613
Atrial fibrillation permanent	26 (25)	23 (16)	9 (15)	10 (6)	<0.001
Thyroid disease	5 (5)	4 (3)	3 (5)	6 (4)	0.827
COPD	16 (15)	12 (9)	6 (10)	11 (7)	0.127
No comorbidity	20 (19)	41 (29)	17 (29)	61 (37)	0.019
Time between diagnosis and sample, yr	0 (0–0)	0 (0–0.2)	0 (0–0.1)	0 (0–0.2)	0.233
NYHA FC I–II/III/IV	13 (12.5)/76 (72.5)/16 (15)	22 (15.5)/108 (76.5)/11 (8)	11 (19)/43 (73)/5 (8)	34 (21)/122 (74)/9 (5)	0.114
					*0.010
6MWD, m	144 (48–288)	240 (96–337)	216 (96–342)	323 (144–408)	<0.001
BNP, ng/L	713 (381–1,177)	210 (134–356)	227 (63–571)	47 (19–95)	<0.001
RAP, mm Hg	13 ± 5	10 ± 5	11 ± 5	8 ± 4	<0.001
mPAP, mm Hg	44 ± 9	45 ± 12	43 ± 10	41 ± 13	0.011
PAWP, mm Hg	14 ± 7	12 ± 5	14 ± 6	12 ± 5	0.030
CI, L/min/m^2^	2.0 ± 0.7	2.4 ± 0.9	2.3 ± 0.9	2.6 ± 0.9	<0.001
PVR, WU	10 ± 5	9 ± 7	9 ± 6	7 ± 4	<0.001
S$\bar{v}$_O_2__, %	61 ± 12	66 ± 9	64 ± 14	70 ± 10	<0.001

*Definition of abbreviations*: 6MWD = 6-minute-walk distance; BNP = brain natriuretic peptide; CI = cardiac index; COPD = chronic obstructive pulmonary disease; CTEPH = chronic thromboembolic PH; mPAP = mean pulmonary arterial pressure; NYHA FC = New York Heart Association functional class; PAWP = pulmonary arterial wedge pressure; PH = pulmonary hypertension; PVR = pulmonary vascular resistance; RAP = right atrial pressure; S$\bar{v}$_O_2__ = mixed venous oxygen saturation; WU = Wood units.

Data are presented as *n* (%), mean ± SD, or median (interquartile range).

* Cluster 1 versus cluster 4.

In the subset of 131 patients with PAH, patients in cluster 4 had the best survival, patients in cluster 1 had the worse survival, and patients in clusters 2 and 3 had a similar survival (log-rank *P* = 0.73; Figure E8). Hence, in the subsequent survival analyses in PAH-only independent cohorts, clusters 2 and 3 were combined.

### Cross-Check with Known Prognostic Biomarkers

To “sense check” our clusters, we compared the plasma levels of previously identified prognostic protein biomarkers (7, 9, 10, 16–20) across the clusters (Figure E9). Many, such as BNP, NT-proBNP, β-NGF (β-Nerve Growth Factor), CXCL9 (C-X-C Motif Chemokine Ligand 9), Activin A, FSTL3 (follistatin-like 3), renin, MMP2 (matrix metalloproteinase 2), TIMP1/TIMP2 (inhibitors of metalloproteinases 1 and 2), TSP2 (thrombospondin 2), IGFBP1 (insulin-like growth factor-binding protein-1), IL1-R4 (interleukin 1–like receptor-4), IL-18, PXDN (peroxidasin), or SVEP1 (polydom), were significantly increased in cluster 1 (poorest survival) compared with the other clusters, the direction of change consistent with previously published observations for these proteins.

### Enrichment of Biological Pathways

To further understand the proteins that characterize each of the clusters (Figure E10), we conducted an enrichment analysis of the top 100 up- and downregulated proteins from each cluster. This highlighted significant biological pathways, revealing a diverse array of enriched terms that provide valuable insights into the underlying molecular mechanisms (Table E9 and Figure E11). For example, extracellular matrix organization proteins were downregulated in cluster 4 (associated with best survival) but upregulated in cluster 1 (worse survival; Figure E11).

### Validation of Proteomic Clustering in Two Independent PAH Cohorts and One Cohort of PH-LHD

We trained a random forest classifier on a combination of 61 proteins, selected by LASSO regression, to assign new samples to one of four clusters. LASSO scores for each cluster (Table E10) clearly distinguished cluster membership (Figure E12). Using these scores as input, we trained a random forest classifier on the discovery cohort and applied this to predict clusters in the independent cohorts (Figure E13). To confirm the robustness and clinical relevance of our clusters, we then assessed risk and outcomes. In separate United Kingdom and French PAH cohorts, 97% and 88%, respectively, of patients classified as cluster 4 were either at low risk or intermediate-low risk of death according to the ESC/ERS 4 strata risk tool (1, 2), whereas 75% and 92% of patients classified as cluster 1 were at intermediate-high or high risk of death (log rank test, *P* < 0.001 in both cohorts).

Consistent with our findings in the discovery cohort, 5-year survival was better in cluster 4, worse in cluster 1, and intermediate in clusters 2 and 3 (log rank test, *P* < 0.001 in both PAH validation cohorts at each time; Figures 3C–3F). This was also observed in a PH-LHD patient group (log rank test, *P* = 0.022; Figure E14A) and in a mixed cohort combining the UK patients with PAH and PH-LHD (log rank test, *P* < 0.001; Figure E14B).

### Patient Migration between Clusters over Time and Survival

**Figure 4.**
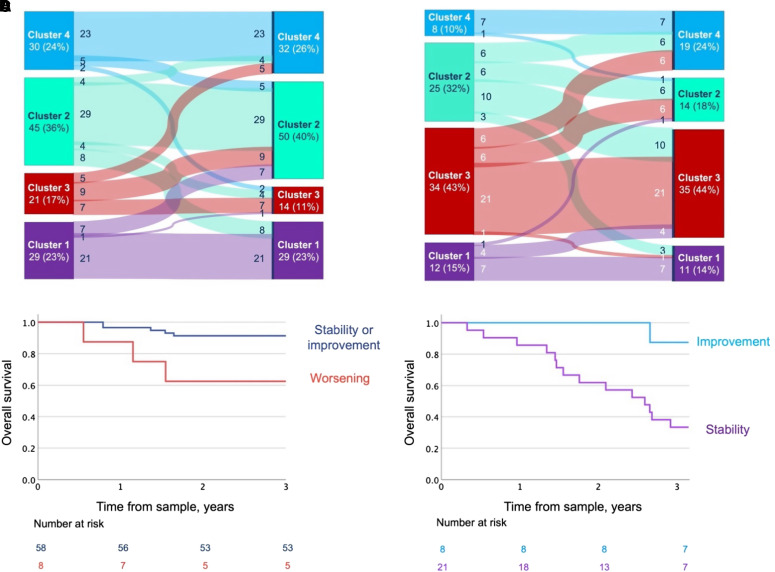
Sankey diagrams showing cluster changes over time in (*A*) UK and (*B*) French cohorts and (*C* and *D*) association with survival. (*A*) In the UK cohort, 36% of patients changed cluster over time. (*B*) In the French cohort, 38% of patients changed cluster over time. (*C*) Survival of UK patients in clusters 2 or 3 according to cluster changes over time (stable or improvement in dark blue, worsening to cluster 1 in red). Log rank test, *P* < 0.001. (*D*) Survival of UK patients in cluster 1 according to cluster changes over time (improvement in light blue, stable in purple). Log rank test, *P* = 0.006.

To assess the dynamic nature of our clusters, we assessed serial samples from the UK PAH Cohort and the French EFORT validation cohorts. In the UK and French cohorts, 36% and 38%, respectively, changed cluster over time (Figures 4A and 4B). Patients who switched from cluster 2 or 3 to cluster 1 (*n* = 8) had a poorer survival than those who remained in the same cluster or switched to cluster 4 (*n* = 58; log rank test, *P* < 0.001; Figure 4C), whereas changes from cluster 1 to another (*n* = 8) were associated with a significant improvement in survival (log rank test, *P* = 0.006; Figure 4D).

### Identification of Potential Theragnostic Biomarkers

**Figure 5.**
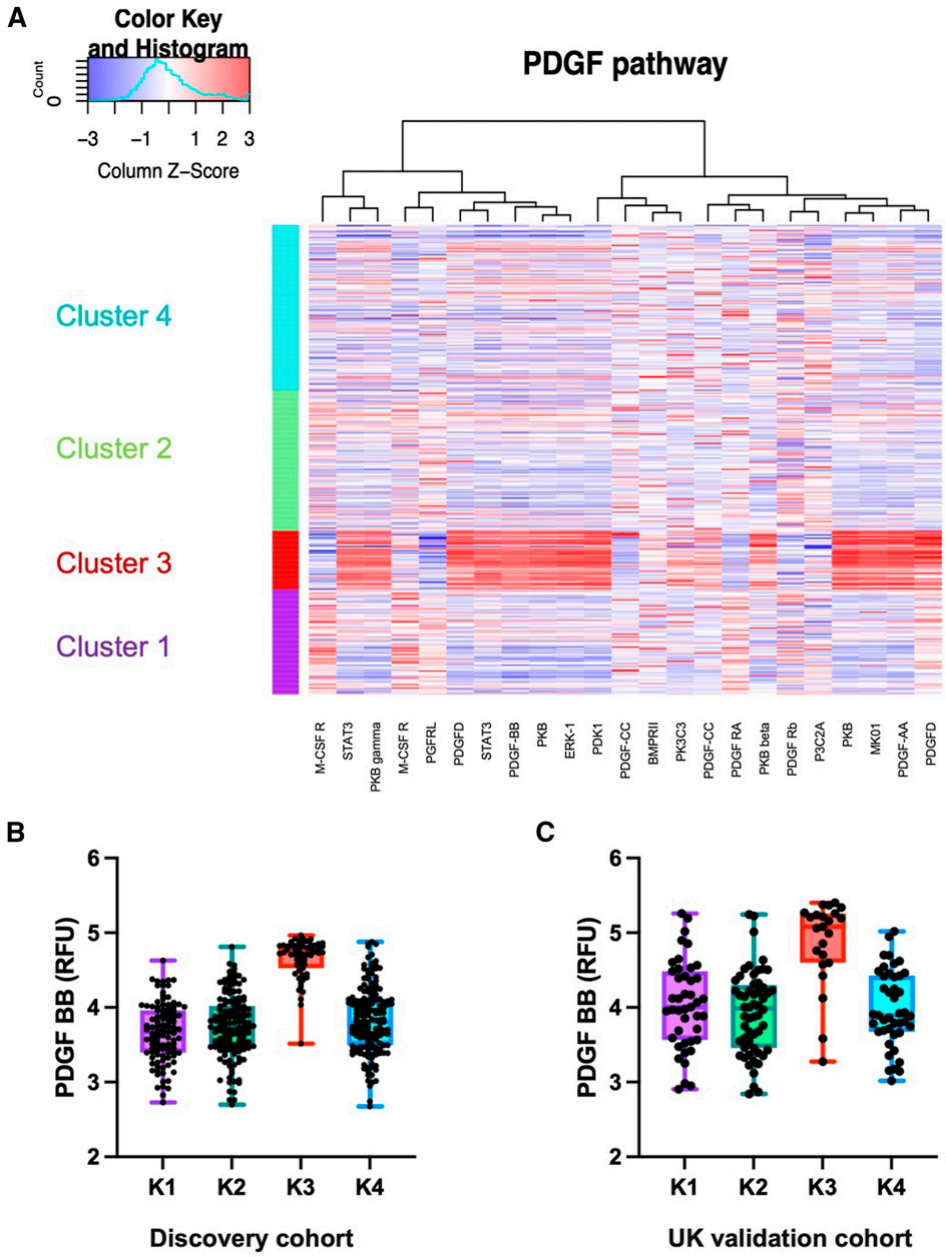
Enrichment of PDGF (Platelet-Derived Growth Factor) pathway in cluster 3. (*A*) Heatmap and (*B* and *C*) levels of PDGF-β-blocker according to clusters in discovery cohort and UK validation pulmonary arterial hypertension cohort. Statistics: (*B*) Nonpaired ANOVA test, *P* < 0.001. All Dunnett’s pairwise comparisons versus cluster 3 (K3), *q* < 0.001. (*C*) Nonpaired ANOVA test, *P* < 0.001. All Dunnett’s pairwise comparisons versus K3, *q* < 0.001. RFU = relative fluorescence unit.

**Figure 6.**
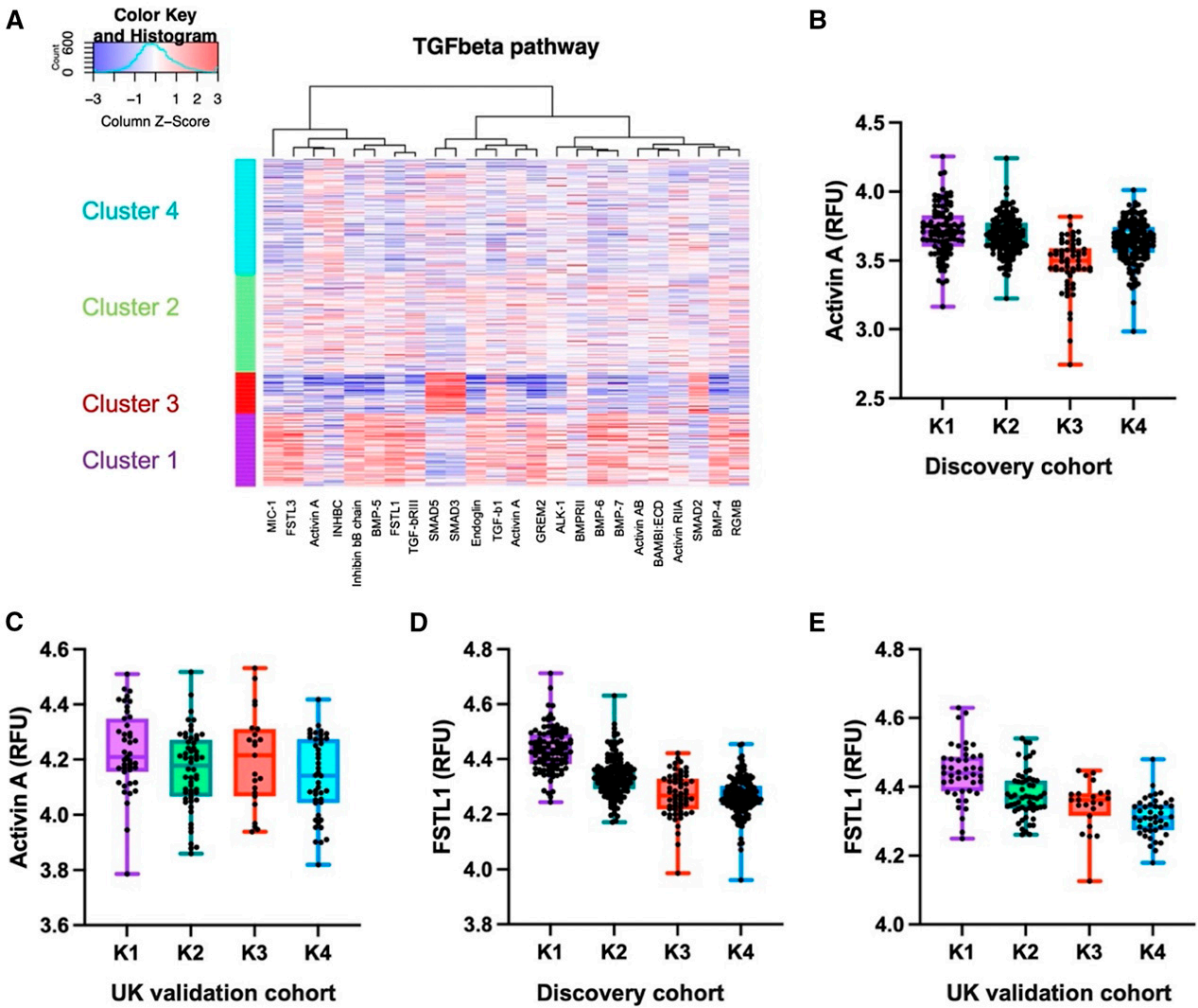
Enrichment of TGF-β pathway cluster 1. (*A*) Heatmap and levels of (*B* and *C*) Activin A and (*D* and *E*) follistatin according to clusters in discovery and UK validation pulmonary arterial hypertension cohorts, respectively. Statistics: (*B*) Nonpaired ANOVA test, *P* < 0.001. Dunnett’s pairwise comparisons cluster 1 (K1) versus K3 and K1 versus K4, *q* < 0.001. (*C*) Nonpaired ANOVA test, *P* = 0.019. Dunnett’s pairwise comparison K1 versus K4, *q* = 0.009. (*D*) Nonpaired ANOVA test, *P* < 0.001. All Dunnett’s pairwise comparisons versus K1, *q* < 0.001. (*E*) Nonpaired ANOVA test, *P* < 0.001. All Dunnett’s pairwise comparisons versus K1, *q* < 0.001. FSTL1 = follistatin; RFU = relative fluorescence unit; TGF-β = Transforming Growth Factor-β.

To investigate the therapeutic relevance of the protein clusters, we investigated two potential disease-modifying drug targets: the PDGF and TGF-β pathways (Figures 5A and 6A). The PDGF pathway was upregulated in cluster 3 compared with other clusters (Figure 5A). In particular, levels of PDGF-BB were higher in cluster 3 than in other clusters in both discovery and validation cohorts (Dunnett’s pairwise comparisons, *P* < 0.001; Figures 5B and 5C).

The TGF-β pathway was downregulated in cluster 3 and upregulated in cluster 1 (Figure 6A). Levels of Activin A were higher in cluster 1 than in clusters 3 and 4 (Dunnett’s pairwise comparisons, *q* < 0.001) in discovery (Figure 6B) and higher than in cluster 4 in the validation cohort (Dunnett’s pairwise comparisons, *q* = 0.009; Figure 6C), whereas levels of follistatin were significantly higher in cluster 1 than in other clusters in both cohorts (ANOVA, *P* < 0.001; Dunnett’s pairwise comparisons, *q* < 0.001; Figures 6D and 6E).

### Distribution of Cluster Proteins in a Population Cohort

The proportion of patients assigned to cluster 1 (highest mortality) fell with decreasing mPAP in the UK discovery cohort (Table E11). The Whitehall II study provided the opportunity to investigate the distribution of the proteins in the general population. We hypothesized that the clusters associated with intermediate-high–risk PH would be poorly detected in this cohort. Of the 6,196 Whitehall II participants with valid protein data, only 2 (0.032% vs. 22.4% in PH) belonged to cluster 1, whereas clusters 2 (*n* = 213; 3.4% vs. 30% in PH), and 3 (*n* = 527; 8.5% vs. 12.6% in PH) were uncommon, and cluster 4 represented the majority (*n* = 5,454; 88% vs. 35% in PH; Figure E15).

## Discussion

Here, a comprehensive analysis of the circulating proteome, involving 470 patients with PH (groups 1–4) and 195 control subjects, dissected the clinical presentation of PH into distinct molecular subsets. We identified plasma proteins that distinguish PH from both healthy and NoPH (disease) control subjects and, through unsupervised clustering independent of the clinical classification, revealed four clusters of patients with PH linked biologically to underlying pathways manifesting significant differences in survival. In doing this, we identified patients in whom the underlying pathology may plausibly be driven by pathways targeted by drugs currently under investigation. These patients could be prioritized for targeted clinical studies.

It is well recognized that PH is a convergent phenotype that presents significant challenges for diagnosis, treatment, and prognosis. The widely used clinical classification acknowledges that PH may arise alone or as a comorbidity but does not inform the underlying pathology. The plasma protein profile can help to differentiate subjects with PAH from healthy control subjects (10) and inform prognosis for patients with PAH (9, 10, 16) but has also emerged as a molecular instrument for unraveling the pathophysiological diversity of PH (10). Sweatt and colleagues used a multiplex immunoassay and machine learning to identify immune endotypes in PAH (7). Here we broaden the proteomic net and examine differences in circulating levels of approximately 7,000 proteins across the clinical spectrum. We were able to identify protein signatures associated with the clinically defined PH groups, but there was significant overlap across these groups. In short, the clinical groups did not distinguish patients based on disturbed biological pathways that would inform treatment. We therefore turned to advanced unsupervised bioinformatics to classify patients with PH based on plasma protein distribution.

We identified four distinct clusters of patients based on their proteomes. The biological importance of these is evident in that they stratified patients with different clinical severity and outcomes. Validation analyses performed on two PAH-only independent cohorts and one cohort of PH-LHD confirmed the link between clusters and survival, emphasizing their clinical relevance, and showed that dynamic changes in clusters over time were associated with significant changes in survival. The distribution of recognized prognostic biomarkers in PAH across the clusters was consistent with previous studies and further underscores their biological significance (7, 9, 10, 16–20). For example, circulating levels of BNP, NT-proBNP, renin, cytokines, Activin A, FSTL3, and proteins involved in extracellular matrix organization were increased in cluster 1 (the cluster with the poorest survival) and lowest in cluster 4 (the cluster with the best survival). This makes biological sense; circulating BNP and NT-proBNP report on cardiac workload, whereas circulating levels of extracellular matrix organization proteins may link to ongoing vascular remodeling (21, 22).

The real clinical opportunity in the four protein clusters is not their use as prognostic markers but their potential to guide therapeutic decision making through the prism of personalized medicine. As proof of principle, we investigated known drug targets: the PDGF and TGF-β pathways (23). The PDGF pathway was upregulated in cluster 3. This pathway has long been implicated in the pathogenesis of PH, because of its role in mediating vascular remodeling and proliferation of pulmonary artery smooth muscle cells (24). Oral imatinib, a tyrosine kinase inhibitor, has been shown to improve hemodynamics and exercise capacity in PAH, although with concerns about safety in this patient group (25). The PDGF pathway remains of active interest as a therapeutic target (26), and cluster 3 could be exploited to identify a subset of patients in whom the benefits of tyrosine kinase inhibition outweigh the potential side effects.

Likewise, upregulation of the TGF-β pathway in cluster 1 might signal a group of patients most likely to benefit from drugs such as the activin ligand trap, sotatercept, that target this pathway. Genetic and now pharmacological studies with sotatercept underscore the importance of the TGF-β pathway in PAH. Its dysregulation has been linked to endothelial dysfunction, inflammation, and fibrosis in the pulmonary vasculature (27, 28). Sotatercept, derived from the activin receptor type IIA, is believed to rebalance BMP (bone morphogenetic protein)–TGF-β signaling in PAH (28). A recent proteomic study of a small number of patients has reported the effect of sotatercept on a panel of circulating biomarkers, including reducing BMP9 and BMP10 levels and changes in inflammatory mediators (29). The Phase II PULSAR and the phase III STELLAR trials have provided evidence that sotatercept, when added to standard therapy, significantly improves hemodynamics and exercise capacity in patients with PAH, although not without safety concerns (30–32). Using the proteomic signature from cluster 1 may permit better targeting of the drug to patients who will benefit.

This introduces the concept of theragnostics to PH medicine: the use of a test to inform and direct drug therapy. Currently, drug selection is based on the clinical subgroup to which a patient is assigned and their “risk score,” an assessment of the severity of their PH (1, 2). By identifying patients with upregulated PDGF or TGF-β pathways, clinicians could tailor PAH management when considering drugs that act on these pathways. Treatments could be directed toward the specific molecular drivers perturbed in each patient and improve the benefit–harm balance that accompanies every drug. The protein clusters may also identify patients assigned to other clinical PH groups (i.e., outside group 1) who might benefit from these drugs and deserve inclusion in clinical trials. Integrating these clusters, derived from proteomic profiling, into future clinical studies is the next step toward validating their translational value and assessing their potential clinical impact.

A significant strength of our study lies in the large patient cohort recruited in PH expert centers and the validation of our findings across two independent PAH cohorts, with serial samples, and one cohort of PH-LHD. Although generated in a cohort of patients with largely prevalent PH (UK cohort), the four protein clusters were reproduced in newly diagnosed, treatment-naive patients (French cohort) and were not affected by duration of illness; the median duration of PH in the discovery cohort was similar across the clusters, so it was not a major factor in determining protein distribution. Conversely, the risk-associated clusters were not prevalent in the general population (Whitehall II study). This observation speaks to the importance of using the four clusters in context, refining the management of patients with a clinical diagnosis of PH.

This study used an mPAP ⩾ 25 mm Hg rather than >20 mm Hg to define PH, in line with the license for currently approved drugs; excluding the small number (*n* = 25) of patients with a mPAP >20 to ⩽24 mm Hg from the analysis did not affect the clusters. There are limitations to the SomaScan assay. Although the platform has a large number of proteins, there remain many more measurable proteins in plasma not included in this analysis. Furthermore, the assay provides measurements as relative fluorescence units, rather than absolute concentrations. These values can be used to compare patients and changes over time, but they are not suitable for use in clinical applications that require absolute concentration to inform treatment decisions. Previous studies showed a good correlation between SomaScan measurements and ELISA (9, 33–35) and mass spectrometry (36), giving this assay a high degree of confidence. Blood samples were collected alongside routine clinical plasma samples, showing the practical deployment of this protein panel in a clinical setting. However, for the panel to be routinely useful and at a reasonable cost, the rapid automated testing of the panel of proteins needed to identify clusters 1 and 3 on a widely available platform would be required.

### Conclusions

Through an unsupervised analysis of the plasma proteome, we have identified molecular signatures that may redefine the classification and management of PH, echoing precision medicine approaches adopted in other fields, such as oncology. We described four PH patient groups linked to underlying pathways, independent of the current clinical classification of PH. The differential expression of PDGF and TGF-β pathways across the proteomic clusters signposts a new era of personalized therapy in PH. These findings advocate for the inclusion of plasma protein profiling in routine clinical assessment to enable the precise targeting of molecular pathways with tailored therapeutics, ultimately improving patient outcomes and advancing the field toward truly personalized medicine.

## Supplemental Materials

10.1164/rccm.202408-1574OC Online Data Supplement
