# Supplementary material for: Clustering Patients with Pulmonary Hypertension Using the Plasma Proteome
Source: Am J Respir Crit Care Med. 2025 May 9;211(8):1492–503. doi: 10.1164/rccm.202408-1574OC (PMC12369885; doi:10.1164/rccm.202408-1574OC)
Supplement: Online Data Supplement [file rccm.202408-1574OCS1.docx]

**Clustering Pulmonary Hypertension Patients Using the Plasma Proteome**

Athénaïs Boucly, MD, PhD, Shanshan Song, PhD, Merve Keles, PhD, Dennis Wang, PhD, Luke S. Howard, MD, DPhil, FRCP, Marc Humbert, MD, PhD, Olivier Sitbon, MD, PhD, Allan Lawrie, PhD, A A Roger Thompson, Philipp Frank, PhD, Mika Kivimaki, FMedSci, Christopher J. Rhodes, PhD, Martin R. Wilkins, DSc FMedSci

**ONLINE DATA SUPPLEMENT**

1. **Extended Methods**

**Participants**

The discovery study population comprised patients with suspected PH who attended a specialist clinic at Imperial College NHS Trust between 2013 and 2021. All patients were managed according to the ESC/ERS guidelines.^12,13^ Patients with PH, defined by a mean pulmonary artery pressure ≥25mmHg, were classified in Group 1 (PAH), Group 2 (PH associated with left heart disease, PH-LHD), Group 3 (PH associated with lung disease, PH-lung) or Group 4 (chronic thrombo-embolic PH, CTEPH).^12,13^ Patients referred with suspected PH but with a mean pulmonary artery pressure <25mmHg on right heart catheterisation were classified as symptomatic disease controls. Contemporaneous plasma samples were obtained from volunteers without cardiovascular or respiratory diseases who acted as health controls. All patients were recruited with informed written consent and local research ethics committee approval (11/LO/0395 and 17/LO/0563).

Separate cohorts of PH patients with serial plasma samples collected over the same time period were used for independent validation: the UK National Cohort Study (NCT01907295); the French EFORT study: Evaluation of Prognostic Factors and Therapeutic Targets in PAH (NCT01185730); and the Sheffield Teaching Hospitals Observational Study of patients with PH, Cardiovascular or Respiratory Disease (18/YH/0441). The Whitehall II study^14^ provided a dataset based on samples collected from a large cohort that were healthy at baseline to understand the behaviour of PH-associated clusters in a population cohort.

**Sample collection and processing**

With the exception of the Whitehall II cohort, patients were sampled non-fasted at their routine clinical appointment visits. All samples were taken from peripheral veins. Serial samples were available in 125 patients from the UK PAH Cohort and 79 patients from the EFORT cohort. The median follow-up time between the first and the second sample was 12.1 (11.0 – 13.5) months in the UK PAH cohort and 4.6 (3.9 – 7.3) months in the EFORT cohort. The EFORT cohort included only newly diagnosed patients with PAH. Patients were therefore treatment naïve at time of first sample. Patients were treated as follows: Calcium channel blockers n=5, oral monotherapy n=33, oral dual therapy n=33, and initial triple combination therapy n=8. Among the 125 patients from the UK PAH Cohort with serial samples, there were only 17 treatment escalation (11 to dual therapy and 6 to triple therapy).

Plasma EDTA samples were stored at -80^o^C and shipped to SomaLogic (Boulder, CO, USA) for SomaScan proteomic analysis. Samples from the discovery cohort were assayed using the 7K platform (comprising 7335 Somamers targeting 7288 human proteins). Proteomic analysis of the 2 independent validation cohorts used the SomaScan version 4 assay (which measures 4979 human Somamers). In Whitehall II, both 7k and 4.0 assays were used. In all studies, technicians were blinded to patient status. Relative fluorescence units were log-10 scale transformed to normalize protein levels prior to analysis.

**Statistical analyses**

1. **Supervised approach to identify PH specific proteins**

Patients and controls from the UK discovery cohort were randomized into training (80%) and replication groups (20%) to adequately power discovery analysis of all proteins and replication of proteins meeting statistical significance. To ensure the reproducibility of the random analyses, the random seed value was fixed using the *set.seed(123)* function.

Principal component analysis was performed to evaluate the variation in protein expression profiles and to identify patterns of variation across the samples. Proteins levels were compared between PH patients and (healthy and No-PH) controls by logistic regression models, correcting for age, sex and principal component outliers (**Figure S1**). Sensitivity analyses were performed to confirm that protein differences were independent of haemolysis (cell-free haemoglobin as a covariate), coagulation factor X, renal function (cystatin C). All comparisons were corrected for multiple testing using Benjamini-Hochberg false discovery rate (FDR). A threshold of q<0.05 was considered statistically significant.

A LASSO approach was applied to all PH-specific proteins (previously identified) to reduce the number of proteins of interest and identify the optimal combination for predicting PH diagnosis. This modeling approach used 10-fold cross-validation, with the regularization parameter (lambda) determined by the lowest error plus 1 standard error (to minimize overfitting), implemented with the *glmnet* R-package.^15^ Similar analyses were performed in the dataset of proteins statistically different between patients with PH and controls to identify the combination of proteins that best reflected PH pathology. Receiver operating characteristic (ROC) analyses of the different protein combinations were performed using the *pROC* R-package in the replication group of our dataset, then compared to the performance of N-terminal pro-brain natriuretic peptide (NT-proBNP) using DeLong test.

1. **Unsupervised approach:**

**B.1:** clustering analysis based on proteomic profile

Proteins able to identify PH patients from both healthy controls and No-PH controls (in models corrected for age, sex, principal component outliers, haemolysis, coagulation Factor X and cystatin C) were taken forward for clustering analysis (**Figures 1 and 2**). The dimensions of the dataset (comprising the previously identified proteins) were reduced via the Uniform Manifold Approximation and Projection (UMAP) method using *UMAP* R-package, and the derived UMAP dimensions were then used for clustering. We used the *NbClust* R-package which determines the optimal number of clusters (based on the proteomic profile) with the highest stability by varying all combinations of number of clusters (from 2 to 10), distance measures, and clustering methods.

**B.2:** Classifying samples based on cluster membership

We classified samples based on the proteome-based clusters. LASSO regression was first performed to reduce the number of proteins needed to define the clusters. A Random forest classifier (*caret* and *randomForest* R-packages) from LASSO scores was trained to predict the cluster membership of new samples and used to classify samples from other cohorts.

**B.3:** Clinical differences between clusters

Demographic and clinical differences between the different clusters were assessed by non-paired ANOVA or Kruskal-Wallis tests according to the data distribution and chi-squared tests. We compared survival of the different clusters by log-rank test, from plasma sampling to death or censoring. Survival status for PH patients was censored on December 31, 2022. Overall survival was represented using the Kaplan–Meier method. To check whether our results were consistent with previous studies^7,9,10,14–18^, we identified biomarkers known to be associated with prognosis in PAH on a volcano plot showing plasma levels of proteins in cluster with the worst survival.

**B.4:** Enrichment analysis

Molecular enrichment analysis was performed using the *WebGestaltR* R-package to identify up-and down-regulated pathways of each cluster. Heatmaps of proteins within pathways of PAH drugs in development were performed using *gplots* and *pheatmap* R-packages. The relative fluorescence of proteins of interest in the different clusters were compared by non-paired ANOVA tests with Dunnett’s multiple pairwise comparisons.

1. **Cluster performance in the general population**

To evaluate the ability of the clusters to identify participants who would develop PH in an initially healthy population, we assessed the cumulative incidence for participants in each cluster during follow-up. After confirming the proportional hazards assumption, we computed hazard ratios and 95% confidence intervals for membership in a cluster compared to absence at baseline and incident PH at follow-up using Cox proportional hazards models adjusted for age, sex, and ethnicity. To quantify the predictive performance of clusters associated with incident PH, we calculated conventional predictive statistics, including sensitivity, specificity, positive predictive value (PPV), and negative predictive value (NPV).

Statistical analysis was performed in R (version 4.3.1) and SPSS (version 29; IBM). Continuous variables are expressed as mean with standard deviation or median (interquartile range (IQR)) according to the data distribution.

An overview of the full methodology is displayed in **Figure 1**.

1. **Supplemental Results**

**Plasma proteome differences between PH and controls**

First we used logistic regression modelling to find 2616 SOMAmers where circulating levels distinguished PH from healthy controls and 293 that distinguished PH from No-PH (FDR q<0.05, **Figure S2A**). Similar analyses were applied to each clinical PH subgroup; specifically, (i) PAH and healthy controls (2637 SOMAmers) or No-PH (1083); (ii) PH-LHD and healthy controls (1971) or No-PH (830); (iii) PH-lung and healthy controls (2010) or No-PH (717); and (iv) CTEPH and healthy controls (2696) or No-PH (711) (**Figure S3**). In sum, one thousand and eight unique SOMAmers were differentially expressed between both healthy and No-PH controls and at least one subgroup of PH, in models corrected for age, sex and principal component outliers (**Figure S1**, **Table S6**). We applied Fisher's exact test to evaluate the statistical significance of the shared proteins between multiple group comparisons (e.g., PAH vs PH-LHD, PAH vs PH-lung, PAH vs CTEPH, PH-LHD vs PH-lung, etc.), with all p-values found to be < 0.001.

**Reducing 1008 proteins to concise sets associated with PH and clinical PH subgroups**

Next we used lasso regression to identify a more concise combination of 25 proteins that differentiated PH from healthy controls and 40 proteins distinguishing PH from No-PH patients in a training group and demonstrated good sensitivity and specificity in recognising PH when applied to the replication cohort (**Table S7**, **Figure S2B and S2C**); AUC: 0.997 (0.989-1.000), p<0.001 vs healthy controls, 0.722 (0.621-0.823), p=0.001 vs No-PH. The diagnostic performance of these protein combinations outperformed NT-proBNP in distinguishing PH from healthy controls (0.913 [0.856-0.970], DeLong test p=0.006, **Figure S2B**) and performed at least as well as NT-proBNP in distinguishing PH from No-PH (AUC NT-proBNP: 0.658 [0.546-0.770], p=0.013; DeLong test=0.206, **Figure S2C**).

A similar analysis was performed using the 1008 SOMAmers differentially expressed between controls and any PH aetiology to identify the main clinical PH groups (Group 1, 2, 3 or 4, **Figure S4**). Lasso regressions to predict PAH, PH-LHD, PH-lung and CTEPH produced models comprised of 17, 35, 40 and 29 SOMAmers, respectively. These models performed well in identifying PH-LHD, PH-lung or CTEPH among patients with PH in the replication cohort: AUC PH-LHD 0.747 (0.609-0.885), p=0.001; AUC PH-lung 0.745 (0.633-0.857), p<0.001; AUC CTEPH 0.768 (0.663-0.872), p=0.005, respectively (**Table S8**, **Figure S5**). The combination of these 3 models was able to identify patients with PAH by elimination (**Figure S6**): AUC 0.684 (0.552-0.815), p=0.007.

**PH prediction by cluster proteins in a population cohort**

The Whitehall II study provided the opportunity to investigate the performance of the proteins used for cluster analysis in the general population. We hypothesised that the clusters associated with intermediate-high risk PH would be poorly detected in this cohort. Of the 6196 Whitehall II participants with valid protein data, only 2 (0.032% vs 22.3% in PH) belonged to cluster 1 while clusters 2 (n = 213, 3.4% vs 30% in PH), and 3 (n = 527, 8.5% vs 12.6% in PH) were uncommon and cluster 4 represented the majority (n = 5454, 88% vs 35% in PH, **Figure S15**). During the mean follow-up of 19.8 years, 57 (0.92%) participants were hospitalised with a diagnosis of PH (ICD10-code I27.0, I27.2, or I27.9). The cumulative hazard of developing PH was higher in cluster 2 than in clusters 3 and 4, with the separation in hazard curves between these groups beginning 7 years after baseline (**Figures S16 and S17**). The age-, sex- and ethnicity-adjusted hazard ratio for individuals in cluster 2 versus other participants was 2.35 (95% CI 0.93–5.93), but predictive capacity was poor (sensitivity 8.8%, specificity 96.6%, PPV 2.3%, NPV 99.1%, **Table S11**).

1. **Supplemental tables**

**Table S1:** **Demographics, clinical and hemodynamic characteristics of patients with PH**

|  | **Pulmonary arterial hypertension,**  **N=131** | **PH associated with left heart disease,**  **N=122** | **PH associated with lung disease,**  **N=93** | **Chronic thromboembolic PH,**  **N=124** |
| --- | --- | --- | --- | --- |
| **Sex Female / Male**, n (%) | 89 (68)  / 42 (32) | 71 (58)  / 51 (42) | 45 (48)  / 48 (52) | 57 (46)  / 67 (54) |
| **Age,** years | 58 $\pm$ 18 | 70 $\pm$ 11 | 65 $\pm$ 12 | 62 $\pm$ 17 |
| **Treatment naïve patients**, n (%) | 74 (56) | 120 (98) | 79 (85) | 106 (85) |
| **Systemic hypertension**, n (%) | 41 (31) | 46 (38) | 27 (29) | 33 (27) |
| **Diabetes mellitus**,  n (%) | 10 (8) | 25 (20) | 24 (26) | 9 (7) |
| **Ischaemic heart disease**, n (%) | 8 (6) | 3 (2) | 6 (6) | 5 (4) |
| **Atrial fibrillation permanent**, n (%) | 7 (5) | 41 (34) | 11 (12) | 9 (7) |
| **Thyroid disease,** n (%) | 6 (5) | 4 (3) | 4 (4) | 4 (3) |
| **No comorbidity**, n (%) | 44 (34) | 32 (26) | 23 (25) | 40 (32) |
| **Subdiagnosis**, n (%)  Idiopathic PAH  Heritable PAH  Drugs associated PAH  CTD  CHD  Portal hypertension  Other | 39 (30)  4 (3)  1 (1)  42 (32)  23 (17)  12 (9)  10 (8) | na | na | na |
| **Time between diagnosis and sample**, years (IQR) | 0 (0 – 0.9) | 0 (0 – 0) | 0 (0 – 0) | 0 (0 – 0) |
| **NYHA FC**  **I-II / III / IV,** n (%) | 27 (21) / 89 (68)  / 15 (11) | 21 (17) / 93 (76)  / 8 (7) | 10 (11) / 69 (75)  / 13 (14) | 22 (18) / 96 (78)  / 5 (4) |
| **6MWD**, m | 306  (120 – 397) | 192  (96 – 336) | 144  (95 – 281) | 288  (144 – 375) |
| **BNP**, ng/L | 127  (46 – 370) | 224  (112 – 468) | 131  (44 – 596) | 139  (51 – 350) |
| **RAP**, mmHg | 9 $\pm$ 4 | 13 $\pm$ 5 | 10 $\pm$ 5 | 9 $\pm$ 5 |
| **mPAP**, mmHg | 47 $\pm$ 12 | 38 $\pm$ 9 | 42 $\pm$ 10 | 42 $\pm$ 12 |
| **PAWP**, mmHg | 10 $\pm$ 3 | 20 $\pm$ 6 | 12 $\pm$ 5 | 11 $\pm$ 3 |
| **Cardiac output**, L/min | 4.1 $\pm$ 1.8 | 4.5 $\pm$ 2.0 | 4.5 $\pm$ 1.5 | 4.6 $\pm$ 1.9 |
| **Cardiac index**, L/min/m^2^ | 2.3 $\pm$ 0.9 | 2.3 $\pm$ 0.9 | 2.4 $\pm$ 0.7 | 2.4 $\pm$ 0.9 |
| **PVR**, WU | 10 $\pm$ 6 | 5 $\pm$ 3 | 8 $\pm$ 4 | 8 $\pm$ 5 |
| **SvO2**, % | 77 $\pm$ 12 | 75 $\pm$ 11 | 68 $\pm$ 17 | 72 $\pm$ 11 |
| **PAH targeted therapies,** n (%)  CCB  Oral monotherapy  Oral dual therapy  Dual therapy including PGI2  Triple therapy  No data | 3 (2)  35 (27)  48 (37)  4 (3)  19 (14)  22 (17) | na | na | na |

**Abbreviations:** CCB: calcium channel blockers; CTD: connective tissue disease; CHD: congenital heart disease; NYHA FC: New York Heart Association functional class; 6MWD: 6-min walk distance; BNP: brain natriuretic peptide; RAP: right atrial pressure; mPAP: mean pulmonary arterial pressure; PAWP: pulmonary arterial wedge pressure; PGI2: prostacyclin analog; PVR: pulmonary vascular resistance; SvO2: mixed venous oxygen saturation. na: not applicable.

**Table S2: Demographics and clinical characteristics of the discovery cohort, subdivided into training (80%) and replication groups (20%)**

|  | **Training**  **N=532** | | | **Replication**  **N=133** | | |
| --- | --- | --- | --- | --- | --- | --- |
|  | **Healthy controls,**  **N=46** | **NoPH controls,**  **N=109** | **Patients with PH, N=377** | **Healthy controls,**  **N=13** | **NoPH controls,**  **N=27** | **Patients with PH, N=93** |
| **Sex Female / Male**, n (%) | 32 (70)  / 14 (30) | 69 (63)  /40 (37) | 201 (53)  / 176 (47) | 9 (69)  / 4 (31) | 18 (67)  / 9 (33) | 61 (66)  / 32 (34) |
| **Age,** years | 47 $\pm$ 12 | 60 $\pm$ 16 | 64 $\pm$ 15 | 45 $\pm$ 13 | 65 $\pm$ 14 | 63 $\pm$ 17 |
| **Systemic hypertension**, n (%) | 0 (0) | 53 (49) | 117 (31) | 0 (0) | 17 (63) | 30 (32) |
| **Diabetes mellitus**,  n (%) | 0 (0) | 13 (12) | 55 (15) | 0 (0) | 3 (11) | 13 (14) |
| **Ischaemic heart disease**, n (%) | 0 (0) | 4 (4) | 21 (6) | 0 (0) | 2 (7) | 1 (1) |
| **Atrial fibrillation permanent**, n (%) | 0 (0) | 11 (10) | 58 (15) | 0 (0) | 4 (15) | 10 (11) |
| **Thyroid disease,** n (%) | 0 (0) | 1 (1) | 13 (3) | 0 (0) | 0 (0) | 5 (5) |
| **COPD,** n (%) | 0 (0) | 6 (6) | 36 (10) | 0 (0) | 3 (11) | 9 (10) |
| **No comorbidity**, n (%) | 46 (100) | 29 (27) | 108 (29) | 13 (100) | 5 (19) | 31 (33) |
| **Aetiology of PH**, n (%)  **PAH**  **PH-LHD**  **PH-lung**  **CTEPH** | na | na | 106 (28)  104 (28)  70 (18.5)  97 (25.5) | na | na | 25 (27)  18 (19)  23 (25)  27 (29) |
| **NYHA FC**  **I-II / III / IV,** n (%) | na | 37 (34)/ 69 (63) / 3 (3) | 66 (18) / 277 (73) / 34 (9) | na | 10 (37) / 17 (63) / 0 (0) | 14 (15) / 72 (77) / 7 (8) |
| **BNP**, ng/L | na | 48  (13 – 146) | 183  (61 – 438) | na | 44  (26 – 117) | 134  (54 – 485) |
| **RAP**, mmHg | na | 8 $\pm$ 5 | 10 $\pm$ 5 | na | 6 $\pm$ 3 | 11 $\pm$ 5 |
| **mPAP**, mmHg | na | 23 $\pm$ 9 | 43 $\pm$ 12 | na | 19 $\pm$ 3 | 42 $\pm$ 12 |
| **PAWP**, mmHg | na | 12 $\pm$ 4 | 12 $\pm$ 6 | na | 12 $\pm$ 3 | 12 $\pm$ 6 |
| **Cardiac output**, L/min | na | 6.6 $\pm$ 2.8 | 4.4 $\pm$ 1.9 | na | 5.2 $\pm$ 1.8 | 4.5 $\pm$ 1.8 |
| **Cardiac index**, L/min/m^2^ | na | 3.4 $\pm$ 1.5 | 2.4 $\pm$ 0.9 | na | 2.5 $\pm$ 0.9 | 2.4 $\pm$ 1.0 |
| **PVR**, WU | na | 1.6 $\pm$ 1.0 | 8.5 $\pm$ 6.0 | na | 1.4 $\pm$ 0.5 | 7.7 $\pm$ 5.0 |
| **SvO2**, % | na | 76 $\pm$ 8 | 65 $\pm$ 11 | na | 74 $\pm$ 4 | 68 $\pm$ 11 |

**Abbreviations:** COPD: chronic obstructive pulmonary disease; NYHA FC: New York Heart Association functional class; 6MWD: 6-min walk distance; BNP: brain natriuretic peptide; RAP: right atrial pressure; mPAP: mean pulmonary arterial pressure; PAWP: pulmonary arterial wedge pressure; PVR: pulmonary vascular resistance; SvO2: mixed venous oxygen saturation; na: not applicable.

**Table S3: Demographics and clinical characteristics of the validation cohorts**

|  | **UK validation cohort of patients with PAH,**  **N=165** | **French validation cohort of incident patients with PAH,**  **N=79** | **UK validation cohort of patients**  **with PH-LHD, N=64** |
| --- | --- | --- | --- |
| **Sex Female / Male**, n (%) | 114 (69) / 51 (31) | 56 (71) / 23 (29) | 40 (62.5) / 24 (37.5) |
| **Age,** years | 51 $\pm$ 16 | 51 $\pm$ 18 | 70 $\pm$ 11 |
| **Aetiology of PAH**, n (%)  **Idiopathic**  **Heritable**  **Anorexigen** | 138 (83.5)  26 (16)  1 (0.5) | 53 (67)  16 (20)  10 (13) | na |
| **Time between diagnosis and sample**, years (IQR) | 3.5 (1.4 – 7.3) | 0 (0 – 0) | 0 (0 – 0) |
| **NYHA FC**  **I-II / III / IV,** n (%) | 70 (42) / 77 (47) /  18 (11) | 30 (38) / 43 (54) /  6 (8) | 12 (19) / 49 (76) /  3 (5) |
| **6MWD**, m | 329 $\pm$ 164 | 345 $\pm$ 143 | 225 $\pm$ 159 |
| **BNP**, ng/L | 57 (25 – 157) | 117 (47 – 290) | na |
| **NT-proBNP**, ng/L | na | na | 1125 (500 – 2254) |
| **RAP**, mmHg | 9 $\pm$ 6 | 8 $\pm$ 5 | 12 $\pm$ 6 |
| **mPAP**, mmHg | 50 $\pm$ 16 | 51 $\pm$ 12 | 37 $\pm$ 12 |
| **PAWP**, mmHg | 11 $\pm$ 4 | 9 $\pm$ 3 | 20 $\pm$ 5 |
| **Cardiac output**, L/min | 4.4 $\pm$ 1.8 | 4.4 $\pm$ 1.2 | 4.6 $\pm$ 1.7 |
| **Cardiac index**, L/min/m^2^ | 2.3 $\pm$ 0.9 | 2.5 $\pm$ 0.6 | 2.3 $\pm$ 0.8 |
| **PVR**, WU | 11 $\pm$ 6 | 10 $\pm$ 4 | 2 $\pm$ 1 |
| **ESC/ERS 4 strata risk status**, n(%)  Low  Intermediate-low  Intermediate-high  High | 42 (25)  62 (38)  45 (27)  16 (10) | 15 (19)  30 (38)  27 (34)  7 (9) | na |
| **PAH targeted therapies,** n (%)  Calcium channel blockers  Oral monotherapy  Oral dual therapy  Dual therapy including PGI2  Triple therapy  No data | 9 (5)  39 (24)  77 (47)  5 (3)  22 (13)  13 (8) | 5 (6)  33 (41)  33 (41)  0  8 (10)  0 | na |

**Abbreviations:** NYHA FC: New York Heart Association functional class; 6MWD: 6-min walk distance; BNP: brain natriuretic peptide; NT-proBNP: N-terminal pro-brain natriuretic peptide; RAP: right atrial pressure; mPAP: mean pulmonary arterial pressure; PAWP: pulmonary arterial wedge pressure; PGI2: prostacyclin analog; PVR: pulmonary vascular resistance; na: not applicable.**Table S4. Characteristics of the Whitehall II cohort (n=6196)**

| **Sex Female / Male**, n (%) | 1775 (28.6) / 4421 (71.4) |
| --- | --- |
| **Age,** years Mean (SD) | 55.7 (6.0) |
| **Ethnicity White / non-White**, n (%) | 5670 (91.5) / 526 (8.5) |
| **Follow-up Time**, years Mean+SD | 19.8 (3.7) |
| **Incidence of PH at follow-up**, n (rate per 10,000 person-years) | 57 (4.6) |

**Table S5: Percentage of variance explained by each principal component**

| **Principal component (PC)** | **Explained variance, %** |
| --- | --- |
| **PC 1** | 16 % |
| **PC 2** | 7 % |
| **PC 3** | 4.4 % |
| **PC 4** | 3.4 % |
| **PC 5** | 2.6% |
| **PC 6** | 2.0 % |
| **PC 7** | 1.6 % |
| **PC 8** | 1.3 % |
| **PC 9** | 1.2 % |
| **PC 10** | 0.9 % |

**Table S6: Differentially expressed proteins between PH and controls**

|  | **PAH** | **PH-LHD** | **PH-lung** | **CTEPH** | **Any form of PH** |
| --- | --- | --- | --- | --- | --- |
| **Versus healthy controls** | 2637 | 1971 | 2010 | 2696 | 3505 |
| **Versus No-PH controls** | 1083 | 830 | 717 | 711 | 2049 |
| **Versus both healthy and No-PH controls** | 538 | 451 | 300 | 351 | **1008** |

**Table S7: Area under curve of ROC analysis testing the performance in training and validation groups of the combination of proteins obtained by lasso regression to identify PH from healthy controls (A) and PH from symptomatic controls (B)**

|  | **AUC** | **Confidence interval** | **p-value** |
| --- | --- | --- | --- |
| 1. **PH versus healthy controls** | | | |
| **Training group** | 1.000 | 1.000 – 1.000 | <0.001 |
| **Validation group** | 0.997 | 0.989 – 1.000 | <0.001 |
| 1. **PH versus symptomatic controls** | | | |
| **Training group** | 0.918 | 0.889 – 0.947 | <0.001 |
| **Validation group** | 0.722 | 0.621 – 0.823 | 0.001 |

**Table S8: Area under curve of ROC analysis testing the performance in training and validation groups of the combination of proteins obtained by lasso regression to identify PAH from other PH (A), PH-LHD from other PH (B), PH-lung from other PH (C) and CTEPH from other PH (D)**

|  | **AUC** | **Confidence interval** | **p-value** |
| --- | --- | --- | --- |
| 1. **PAH versus other PH** | | | |
| **Training group** | 0.851 | 0.807 – 0.894 | <0.001 |
| **Validation group** | 0.625 | 0.497 – 0.752 | 0.067 |
| 1. **PH-LHD versus other PH** | | | |
| **Training group** | 0.910 | 0.876 – 0.944 | <0.001 |
| **Validation group** | 0.747 | 0.609 – 0.885 | 0.001 |
| **(C) PH-lung versus other PH** | | | |
| **Training group** | 0.961 | 0.942 – 0.980 | <0.001 |
| **Validation group** | 0.745 | 0.633 – 0.857 | <0.001 |
| **(D) CTEPH from other PH** | | | |
| **Training group** | 0.884 | 0.845 – 0.922 | <0.001 |
| **Validation group** | 0.768 | 0.663 – 0.872 | 0.005 |

**Table S9: Enrichment analysis showing significantly up- or down-regulated pathways depending on clusters.**

|  |  | **Pathway** | **Proteins** | **Enrichment ratio** | **FDR**  (or p-value *) |
| --- | --- | --- | --- | --- | --- |
| **CLUSTER 1 (vs other clusters)** | **UP REGULATION** | BMP signalling pathway | BMP4, BMP5, BMP6, FSTL1, FSTL3, GDF15, GREM2, ROR2 | 6.6 | 0.024 |
|  |  | extracellular matrix organization | collagen, cystatin C, fibulin 5, FLRT2, GAS6, MFAP4, MMP2, PRSS2, PXDN, TIMP1, TIMP2, TNC, TNFRSF1A | 5 | <0.001 |
|  |  | Response to growth factor | ANGPT2, BMP4, BMP5, BMP6, EPHA2, FGF23, FLRT2, FSTL1, FSTL3, GAS1, GAS6, GDF15, GREM2, LTBP4, NRP1, ROR2, TNC, VEGFD | 2.9 | 0.024 |
|  | **DOWN REGULATION** | cell-cell adhesion mediated by cadherin | cadherin 3, cadherin 7, plasminogen, serpin F2, WNT3A | 12.7 | 0.042 |
|  |  | negative regulation of blood coagulation | Factor XI, kallikrein B1, plasminogen, protein kinase cGMP-dependent 1, SERPINF2, vitronectin | 9.4 | 0.042 |
| **CLUSTER 4 (vs other clusters)** | **UP REGULATION** | reverse cholesterol transport | APOA1, APOA5, APOM, LIPG | 23.8 | 0.003 |
|  |  | negative regulation of blood coagulation | FII, FXI, KLKB1, KNG1, PLG, PROC, SERPINF2 | 11.9 | <0.001 |
|  |  | protein activation cascade | APCS, C8G, CFHR5, CPN2, FXI, FXIIIB, FII, FVII, FCN2, FCN3, KLKB1, KNG1 | 11.8 | <0.001 |
|  |  | blood coagulation | FXI, FXIIIB, FII, FVII, KLKB1, KNG1, PLG, PROC, SERPINA10, SERPIND1, SERPINF2, SHH, WNT3A | 5 | <0.001 |
|  | **DOWN REGULATION** | extracellular matrix organization | ADAMTSL2, CCDC80, collagen, cystatin C, fibulin, limican, MFAP4, MMP2, NID1, PXDN, TGF beta, TIMP1, TNC, VWF | 5.8 | <0.001 |
| **CLUSTER 2 (vs cluster 3)** | **UP REGULATION** | cell-cell adhesion via plasma-membrane adhesion molecules | ADGRL3, AMIGO1, AMIGO2, CADM1, CDH5, EFNA5, L1CAM, PTPRD, ROBO2, SLITRK1 | 4.5 | 0.014 |
|  |  | cell morphogenesis involved in differentiation | AMIGO1, ANTXR1, collagen, ephrin, fibulin 1, FLRT2, ISLR2, L1CAM, MERTK, NEO1, NRXN3, NRTK2, PTPRD, ROBO2, SEMA4C, SEMA6B, SLITRK1 | 3.5 | <0.001 |
|  |  | regulation of cell development | CDH5, EFNA5, ENG, FBLN1, FLRT2, HSPA5, IL6ST, ISLR2, JAG1, L1CAM, NOTCH3, NTRK2, PRTG, PTPRD, ROBO2, SEMA4C, SEMA6B, SLITRK1, TIMP2 | 3.0 | 0.003 |
|  | **DOWN REGULATION** | positive regulation of cellular protein catabolic process | CSNK2A1, IFNG, MAPK9, MDM2, METTL3, OAZ1, PAFAH1B2, PTEN, RNF41, TNFAIP3, UBE2V2 | 3.9 | 0.244  * p-value <0.001 |

**Table S10: Coefficients obtained by lasso regression to predict the 4 clusters**

|  | **proteins** | **Cluster 1** | **Cluster 2** | **Cluster 3** | **Cluster 4** |
| --- | --- | --- | --- | --- | --- |
|  | **(Intercept)** | -70.12417677 | -9.442547021 | 40.36921283 | 39.19751096 |
| 1 | **HXK4** | -1.093944806 | -2.619288397 | 4.961869077 | -1.248635874 |
| 2 | **Carbonyl reductase 3** | -1.000018282 | -0.294442589 | 2.759713087 | -1.465252216 |
| 3 | **PLOD3** | 0.197246903 | -0.891189542 | 1.339825578 | -0.645882939 |
| 4 | **C9** | 0.152032843 | -0.833053864 | 0.409167888 | 0.271853133 |
| 5 | **SDF-1** | 0.314415507 | 0.225576128 | 0.159494638 | -0.699486273 |
| 6 | **IL-1 R4** | 0.253897789 | -0.174672787 | 0.079731719 | -0.158956722 |
| 7 | **SAA2** | 0.067860448 | -0.015931861 | 0.026830666 | -0.078759253 |
| 8 | **P4R3A** | 0.166896106 | 0.017578666 | 0.023026026 | -0.207500798 |
| 9 | **CRP** | 0.008523857 | -0.006136064 | -0.001318054 | -0.00106974 |
| 10 | **PTGD2** | 0.000108883 | 0.000435364 | -0.002415925 | 0.001871678 |
| 11 | **MCTS1** | -0.005203563 | -0.00012525 | -0.005568738 | 0.010897551 |
| 12 | **PRS57** | 0.034110627 | -0.036287109 | -0.009138822 | 0.011315303 |
| 13 | **COLL1** | 0.417512564 | -0.387378905 | -0.012414183 | -0.017719476 |
| 14 | **Pancreatic alpha-amylase** | -0.04526405 | -0.160487476 | -0.01350491 | 0.219256436 |
| 15 | **SCUB3** | 0.028035874 | -0.078500269 | -0.021834819 | 0.072299214 |
| 16 | **MIC-1** | 0.070248575 | -0.008041477 | -0.030990883 | -0.031216215 |
| 17 | **WIF-1** | 0.058596773 | -0.069371699 | -0.032656765 | 0.043431691 |
| 18 | **Pseudocholinesterase** | -0.288041941 | -0.258408037 | -0.040651767 | 0.587101745 |
| 19 | **MFAP4** | 0.074109492 | 0.02324122 | -0.040868833 | -0.056481878 |
| 20 | **STX2** | 0.088011071 | -0.034635721 | -0.040873097 | -0.012502253 |
| 21 | **ihh** | 0.00777366 | -0.018522615 | -0.051750724 | 0.062499679 |
| 22 | **sICAM-5...79** | 0.071545369 | -0.012818977 | -0.057420246 | -0.001306146 |
| 23 | **SP-B** | 0.064153294 | -0.08918026 | -0.063727148 | 0.088754114 |
| 24 | **Trypsin 2** | 0.534067209 | -0.357763949 | -0.081978623 | -0.094324637 |
| 25 | **IL27B** | 0.521925626 | -0.047251124 | -0.08732443 | -0.387350071 |
| 26 | **PIGR** | 0.115752563 | 0.03050013 | -0.094619794 | -0.051632899 |
| 27 | **Carbonic anhydrase 6** | -0.115774898 | 0.082524547 | -0.099647494 | 0.132897846 |
| 28 | **SVEP1:EGF-like domains 4-6** | 0.778614194 | -0.080413449 | -0.113993999 | -0.584206746 |
| 29 | **KERA** | 0.247380902 | 0.125440345 | -0.114622735 | -0.258198512 |
| 30 | **NOTUM** | -0.09752195 | -0.162506915 | -0.127418277 | 0.387447141 |
| 31 | **sFRP-3** | 0.179402658 | -0.055105606 | -0.132530549 | 0.008233497 |
| 32 | **SVEP1:Sushi 15-18** | 1.631305193 | -0.317140341 | -0.185443215 | -1.128721638 |
| 33 | **Carbohydrate sulfotransferase 9** | 0.595472791 | 0.262609246 | -0.233050014 | -0.625032023 |
| 34 | **SLPI** | 0.807227777 | -0.14206239 | -0.25105336 | -0.414112027 |
| 35 | **fibulin 5** | 0.649853495 | 0.278963647 | -0.301570431 | -0.627246712 |
| 36 | **Sonic Hedgehog** | 0.066721589 | -0.004252337 | -0.311825488 | 0.249356236 |
| 37 | **EDIL3** | 0.202192361 | 0.122287736 | -0.333107056 | 0.00862696 |
| 38 | **GRIA4** | 0.14895679 | -0.208333971 | -0.335373456 | 0.394750637 |
| 39 | **N-terminal pro-BNP** | 1.5558349 | 0.906237434 | -0.342232952 | -2.119839382 |
| 40 | **BNP** | 2.026677573 | -0.068351798 | -0.360443845 | -1.59788193 |
| 41 | **Protein C** | -0.609650247 | 0.445932532 | -0.368240213 | 0.531957928 |
| 42 | **Periostin** | 0.427822973 | -0.058337169 | -0.385847047 | 0.016361242 |
| 43 | **CECR1** | 0.078223196 | 0.66946445 | -0.390114611 | -0.357573035 |
| 44 | **OLFL3** | 0.943687694 | 1.207822597 | -0.396766527 | -1.754743764 |
| 45 | **BMP-6** | 1.004819956 | -0.419617583 | -0.426007198 | -0.159195174 |
| 46 | **NOE1** | 0.505541953 | 0.468634054 | -0.428600362 | -0.545575645 |
| 47 | **sTREM-1** | 0.347291105 | -0.025559276 | -0.494846282 | 0.173114453 |
| 48 | **HE4** | 0.828182948 | -0.149793551 | -0.502992997 | -0.1753964 |
| 49 | **Kininogen, HMW, Two Chain** | -0.233245218 | -0.451978749 | -0.509006116 | 1.194230083 |
| 50 | **ADH4** | -0.429409046 | 0.953535753 | -0.652890521 | 0.128763815 |
| 51 | **ROBO2** | 0.277919383 | 0.377592754 | -0.659314277 | 0.003802139 |
| 52 | **ADH1A** | -0.485196197 | 0.958908421 | -0.699449911 | 0.225737687 |
| 53 | **PTK7** | 1.200335383 | 0.176785572 | -0.737164355 | -0.6399566 |
| 54 | **IGFBP-7** | 1.978137564 | 0.163065896 | -0.75759166 | -1.383611801 |
| 55 | **ANTR1** | 0.221243407 | 0.434781808 | -0.923515748 | 0.267490532 |
| 56 | **ST4S6** | 1.168384486 | -0.393775955 | -0.996157803 | 0.221549272 |
| 57 | **CILP2** | -0.142434885 | 0.523402066 | -1.047874983 | 0.666907802 |
| 58 | **Kininostatin** | -0.022912001 | 0.337024541 | -1.19937035 | 0.88525781 |
| 59 | **KREM1** | 0.857073398 | 0.835584014 | -1.694454484 | 0.001797072 |
| 60 | **Cathepsin S** | 1.118764166 | 1.167394595 | -1.90898098 | -0.377177781 |
| 61 | **RIR2** | 0.899176846 | 0.570903086 | -2.059275513 | 0.589195581 |

61 Somamers with non-zero coefficients were selected from the 123 somamers entered in the lasso regression.**Table S11: Distribution of clusters according to mPAP threshold in discovery cohort**

1. mPAP ≥25 mmHg (n=470)*

Cluster 1 (poor survival): n=105 (22.4%)

Cluster 2: n=141 (30%)

Cluster 3: n=59 (12.6%)

Cluster 4 (best survival): n=165 (35%)

1. NoPH controls with mPAP >20 to ≤24 mmHg (n=25)

Cluster 1 (poor survival): n=2 (8%)

Cluster 2: n=7 (28%)

Cluster 3: n=4 (16%)

Cluster 4 (best survival): n=12 (48%)

1. NoPH controls with mPAP ≤20 mmHg (n=111)

Cluster 1 (poor survival): n=4 (3.5%)

Cluster 2: n=21 (19%)

Cluster 3: n=12 (11%)

Cluster 4 (best survival): n=74 (66.5%)

1. Healthy controls (N=59)

Cluster 3: 1 (2%)

Cluster 4 (best survival): 58 (98%)

* No significant difference (Chi-squared test) between (A) mPAP ≥25mmHg vs (B) mPAP >20 to ≤24 mmHg, p= 0.31; (A) mPAP ≥25mmHg vs (C) NoPH controls with mPAP≤20mmHg, p<0.001.

1. **Supplemental figures**

**Figure S1: Methodology used to select somamers used to distinguish each PH subgroup from the others**

**
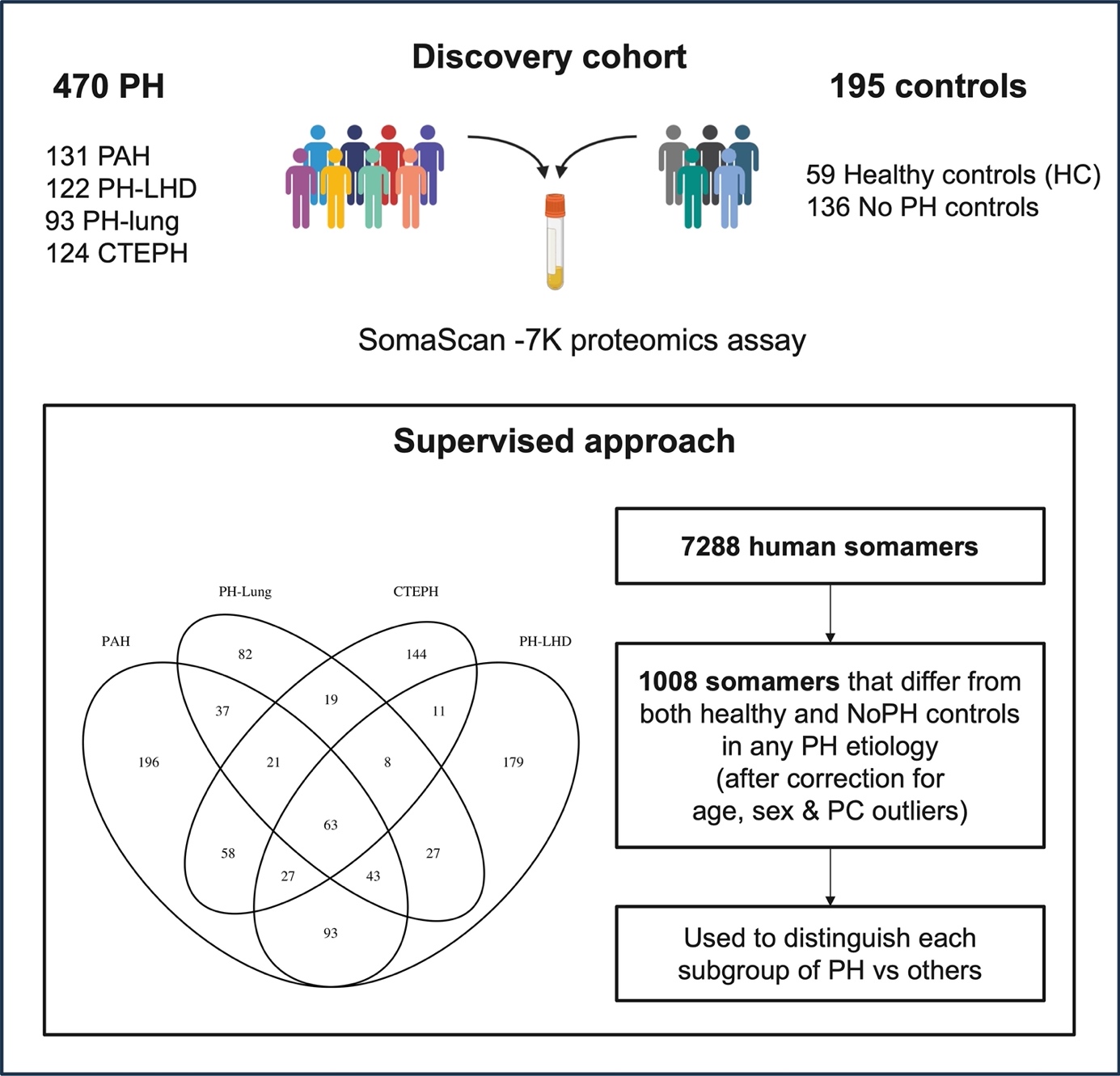
**

**Abbreviations:** PH: pulmonary hypertension; PAH: pulmonary arterial hypertension; PH-LHD: PH associated with left heart disease; PH-lung: PH associated with lung disease; CTEPH: chronic thrombo-embolic PH; HC: healthy controls; No PH: symptomatic disease controls without PH; PC: principal component.

**Figure S2: double Volcano plot showing the proteins differentially expressed in pulmonary hypertension and controls (healthy controls and symptomatic controls) (A) and ROC curves testing the performance in replication group of the combination of proteins obtained by lasso regression to identify PH vs healthy controls (B) and PH vs symptomatic controls (C)**


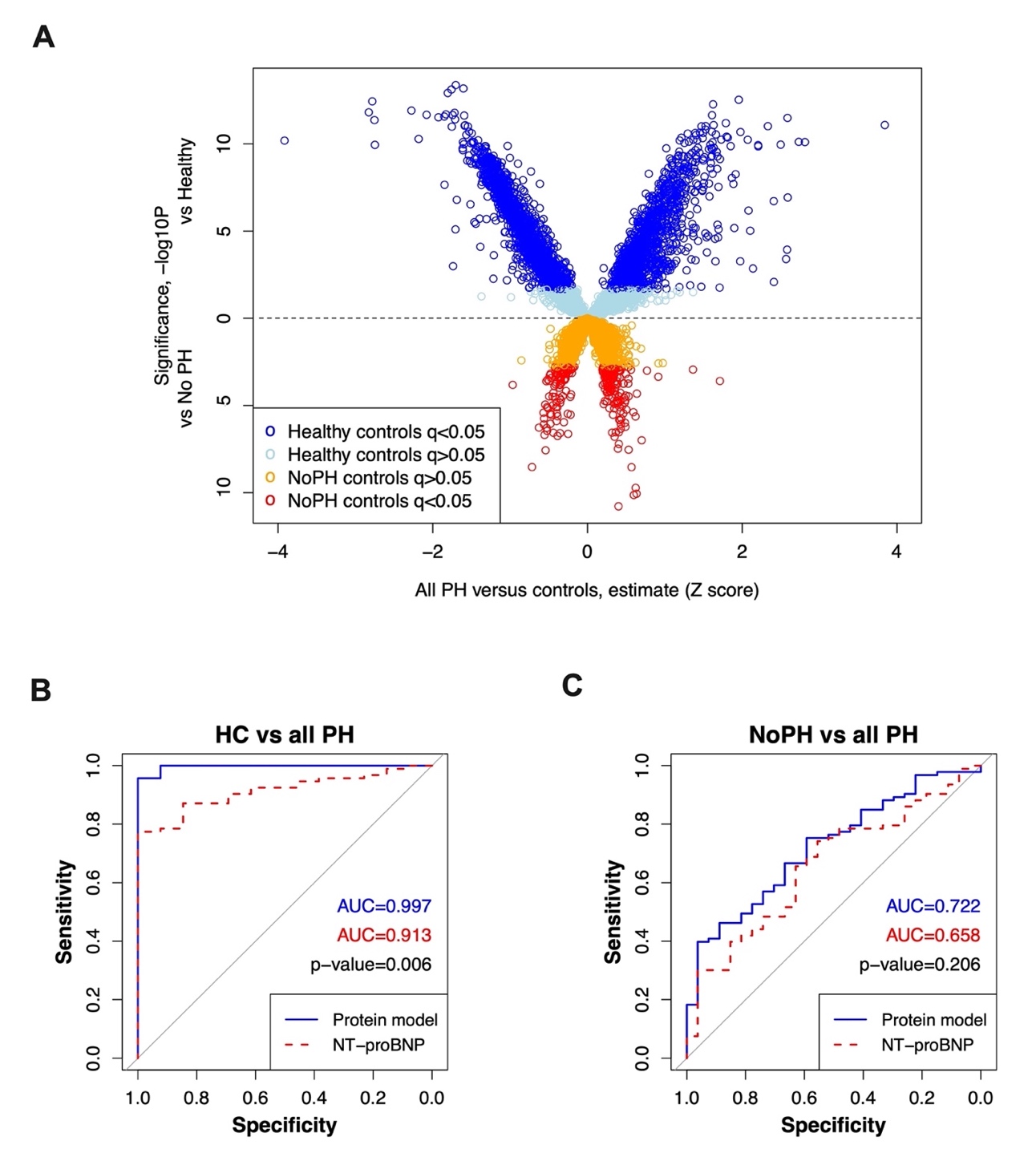


A/ -log10 p value derived from linear regression analysis

B/ AUC combination of 25 proteins: 0.997 (0.989-1.000), p<0.001

AUC NT-proBNP: 0.913 (0.856-0.970), p<0.001; Delong test = 0.006.

C/ AUC combination of 40 proteins: 0.722 (0.621-0.823), p=0.001

AUC NT-proBNP: 0.658 (0.546-0.770), p=0.013; Delong test = 0.206.

**Figure S3: Double Volcano plot showing the proteins differentially expressed in each group of pulmonary hypertensions (A: PAH, B: PH-LHD, C: PH-lung, D: CTEPH) vs controls (healthy controls and symptomatic controls)**

**
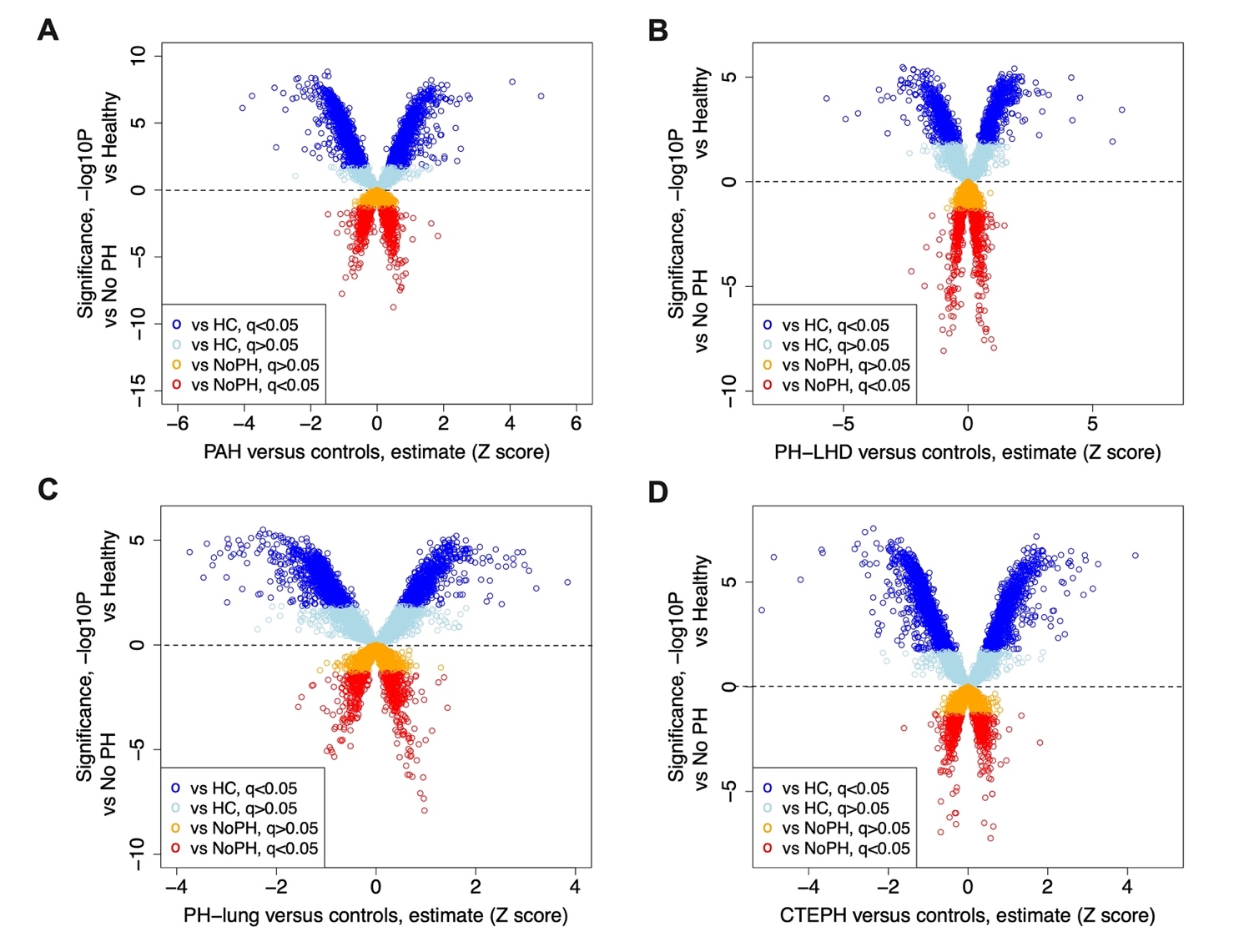
**

A, B, C, D/ -log10 p value derived from linear regression analysis

**Figure S4: Volcano plot showing the proteins differentially expressed by each group of pulmonary hypertension (PH) (A: PAH, B: PH-LHD, C: PH-lung, D: CTEPH) compared to patients with another aetiology of PH
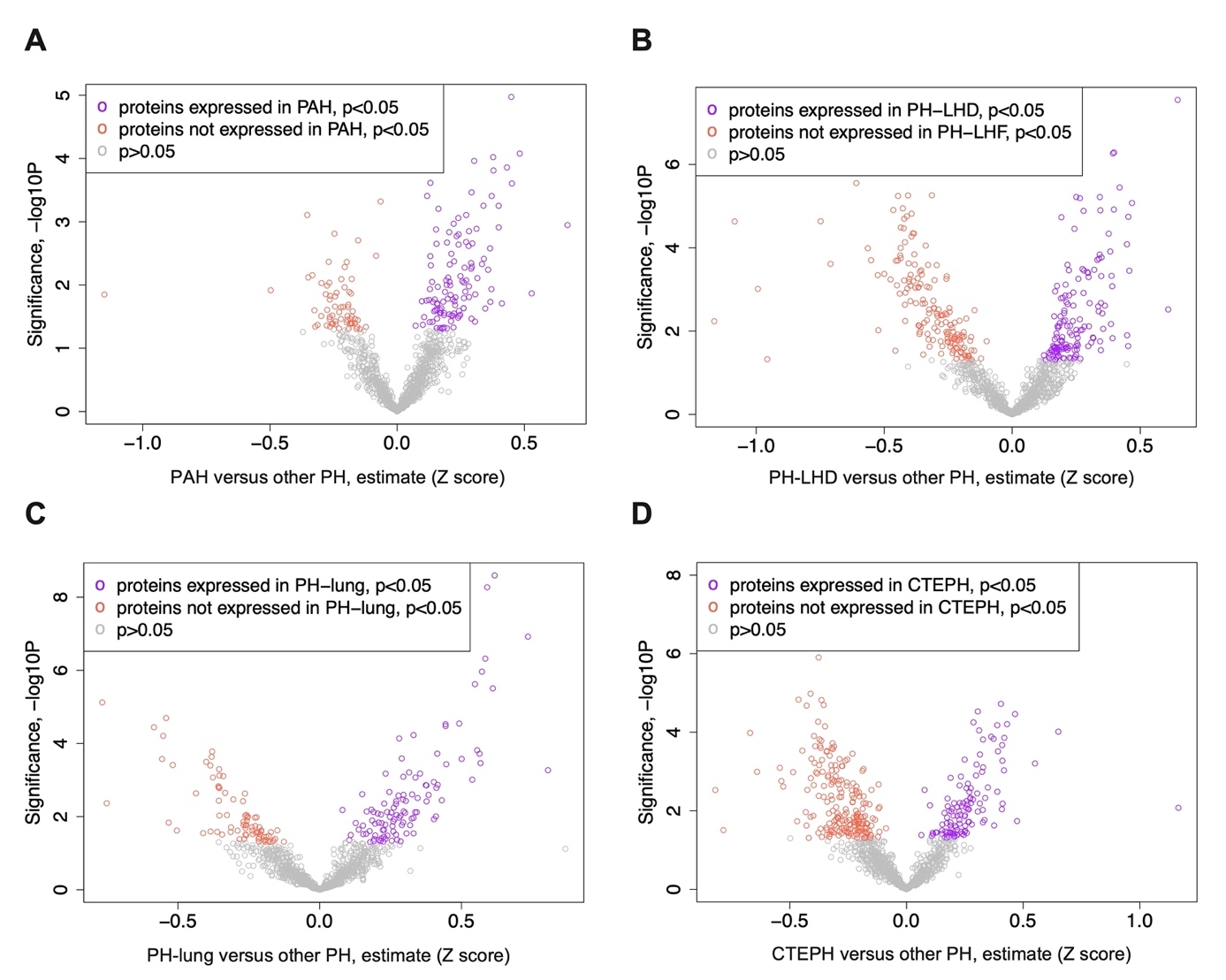
**

A, B, C, D/ -log10 p value derived from linear regression analysis**Figure S5: ROC curves testing the** **performance in replication group of the combination of proteins obtained by lasso regression and NT-proBNP to identify PAH from other PH (A), PH-LHD from other PH (B), PH-lung from other PH (C) and CTEPH from other PH (D)**


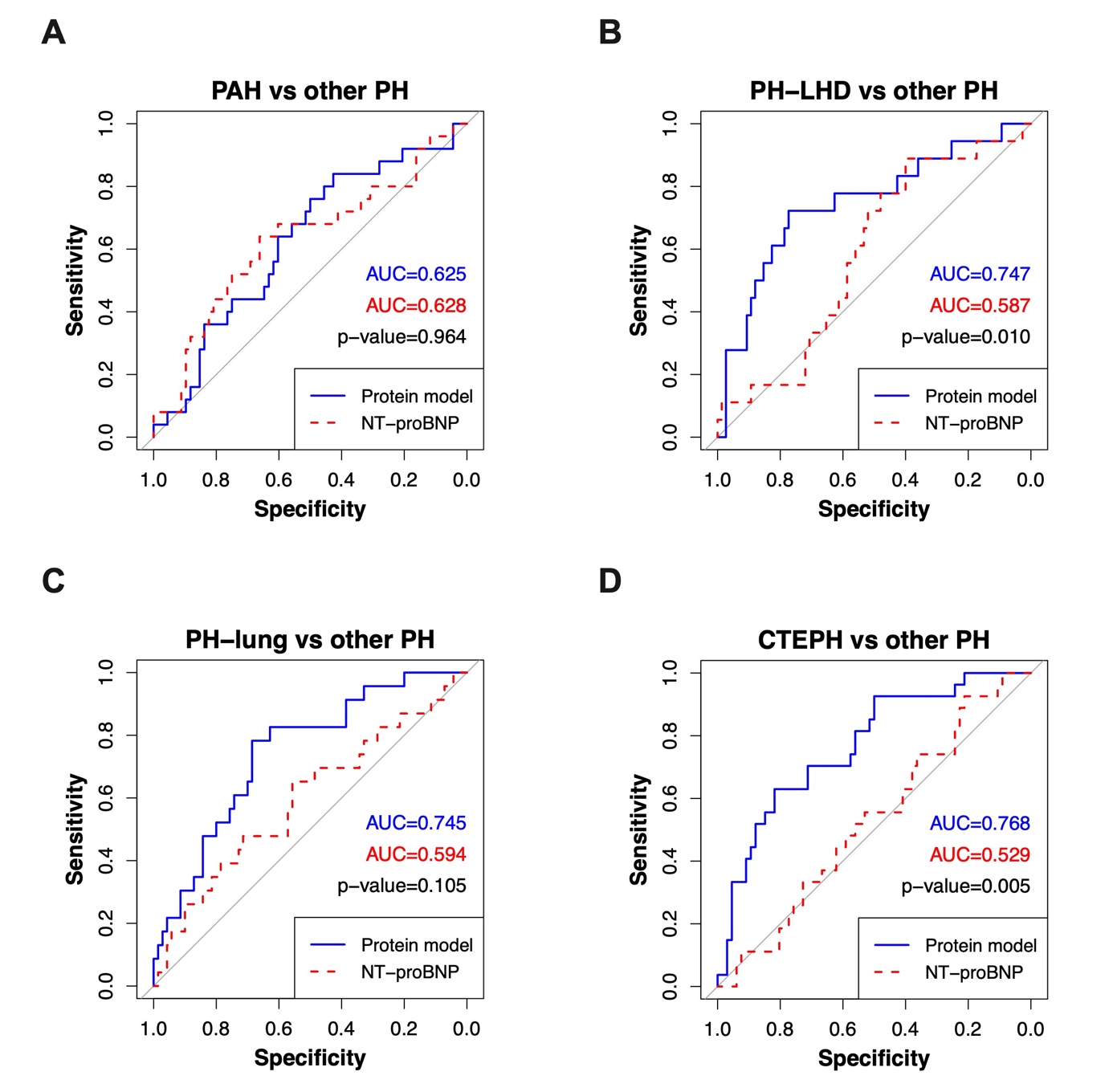


A/ AUC combination of 17 proteins (in blue): 0.625 (0.497-0.752), p=0.067

AUC NT-proBNP (in red): 0.628 (0.491-0.765), p=0.059; Delong test = 0.964.

B/ AUC combination of 35 proteins (in blue): 0.747 (0.609-0.885), p=0.001

AUC NT-proBNP (in red): 0.587 (0.449-0.724), p=0.257; Delong test = 0.010.

C/ AUC combination of 40 proteins (in blue): 0.745 (0.633-0.857), p<0.001

AUC NT-proBNP (in red): 0.594 (0.454-0.735), p=0.177; Delong test = 0.105.

D/ AUC combination of 29 proteins (in blue): 0.768 (0.663-0.872), p=0.005

AUC NT-proBNP (in red): 0.529 (0.403-0.654), p=0.669; Delong test = 0.005.

**Fig S6: ROC curve of the logistic regression of the combination of proteins able to identify groups 2, 3 and 4 PH in order to test the ability to identify PAH from other PH groups**

AUC combination of models: 0.684 (95% CI: 0.552 – 0.815), p=0.007

**Figure S7: Heatmap of 165 somamers used to identify clusters in the discovery cohort**

**Figure S8: Kaplan-Meier survival curves according to clusters in patients with PAH in the discovery cohort**

**
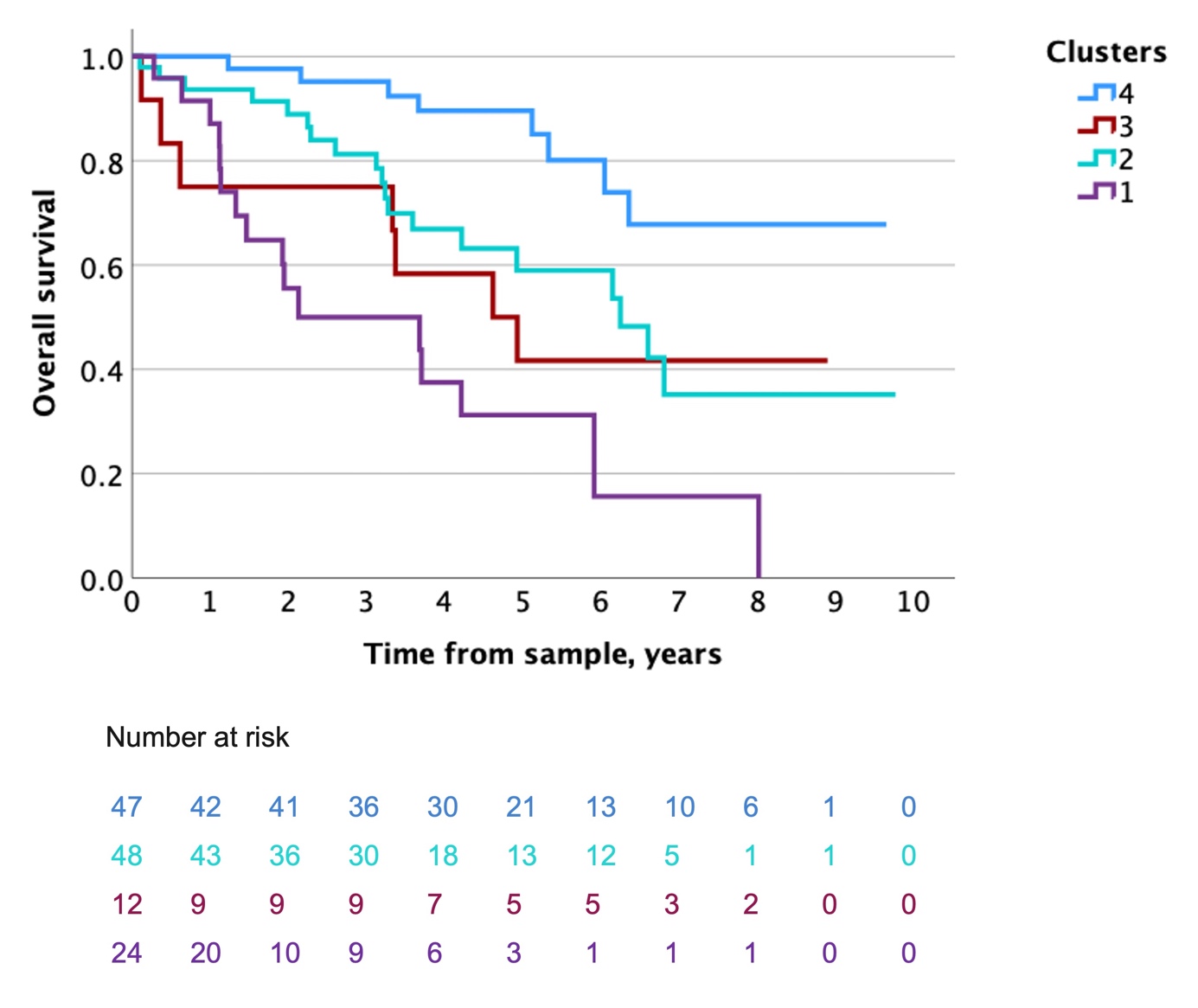
**

Log rank test clusters 2 versus 3, p=0.73.

**Figure S9: Volcano plot showing plasma levels of proteins, including known prognostic biomarkers, in cluster 1 compared to other clusters**

A, B, C, D/ -log10 q value derived from linear regression analysis

**Figure S10: Volcano plot showing up and down-regulated proteins in cluster 1 (A) cluster 4 (B) and cluster 2 (C)**

**
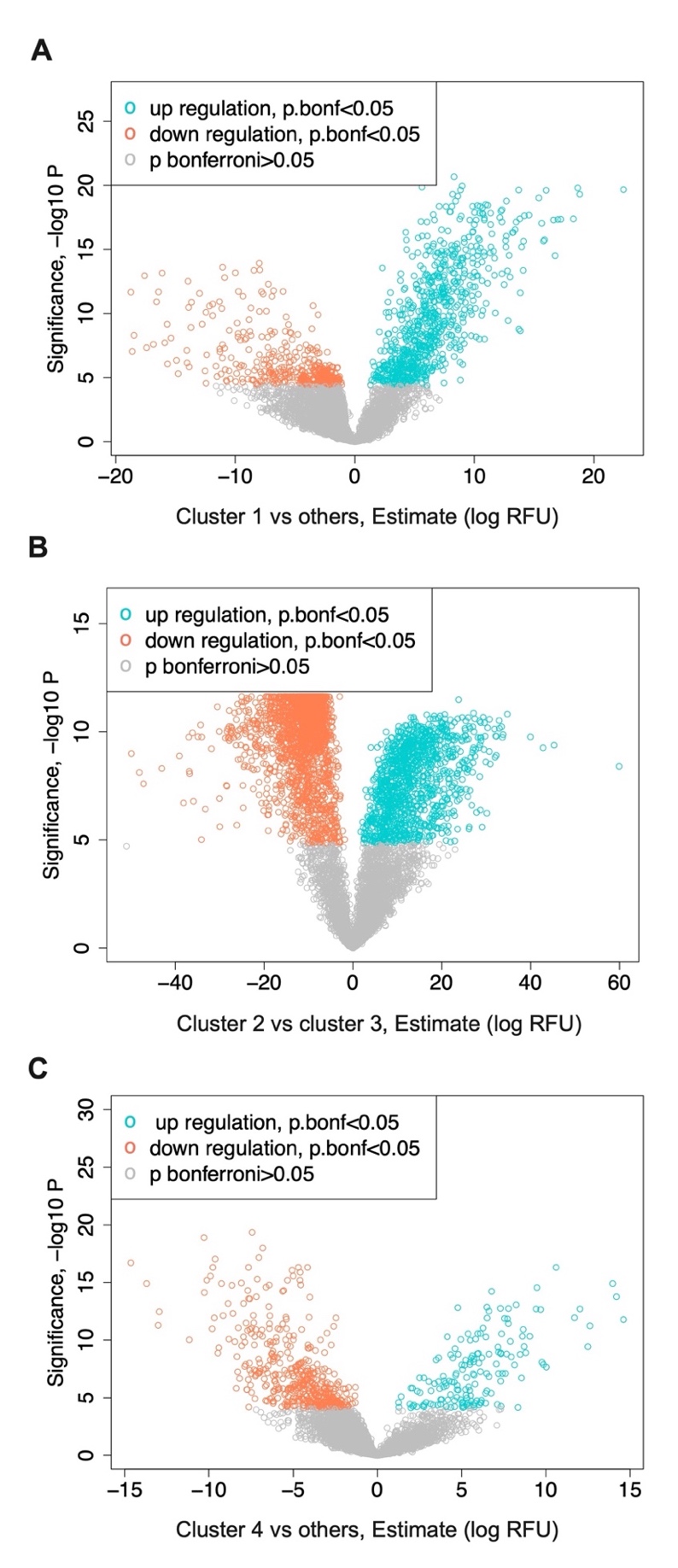
**

A, B, C/ -log10 p value derived from linear regression analysis

**Figure S11: Enrichment analysis of the top 100 up- and down-regulated proteins in cluster**

**
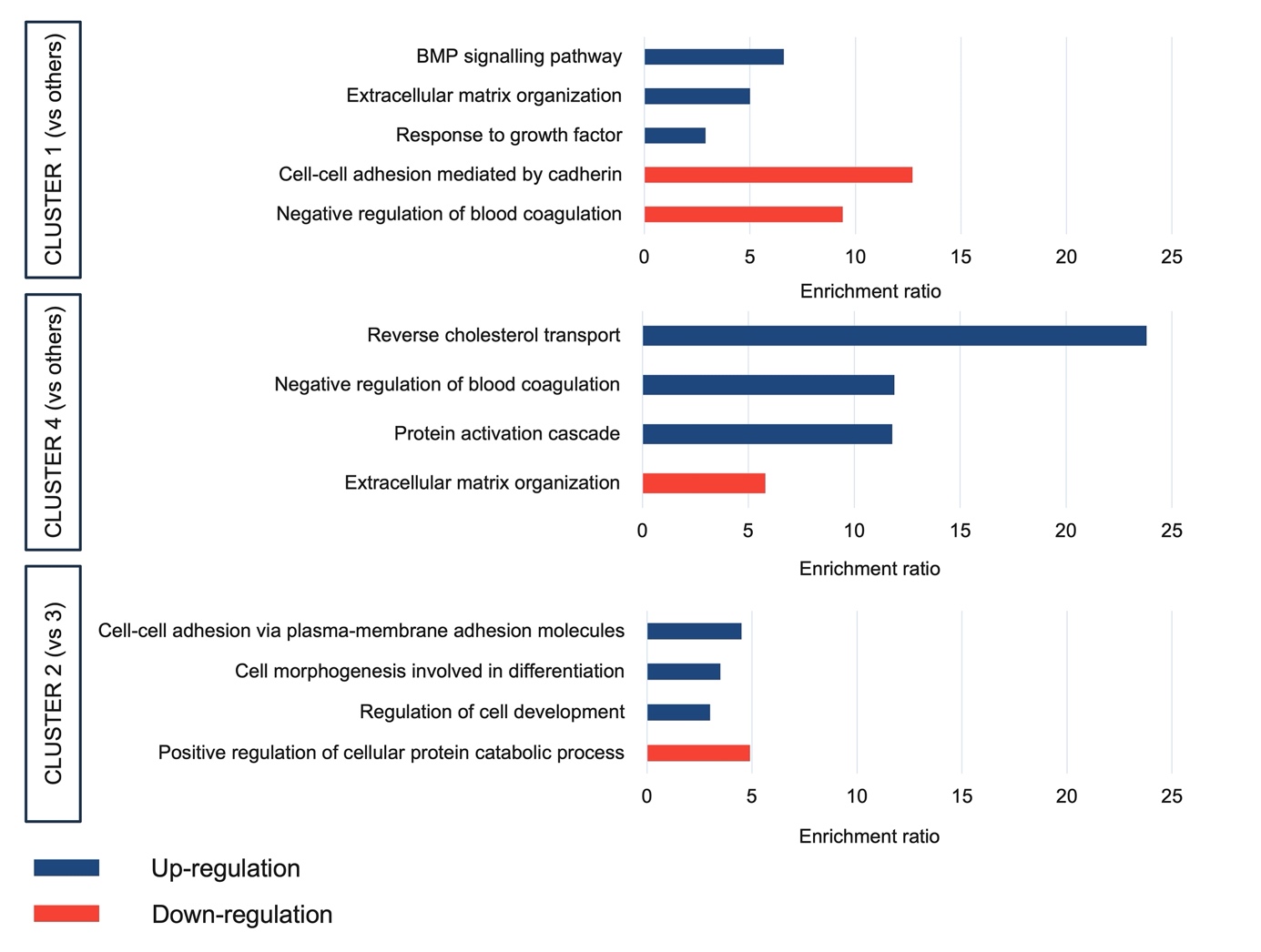
**

**Figure S12: Lasso scores of each cluster in the discovery cohort of patients with pulmonary hypertension**

**
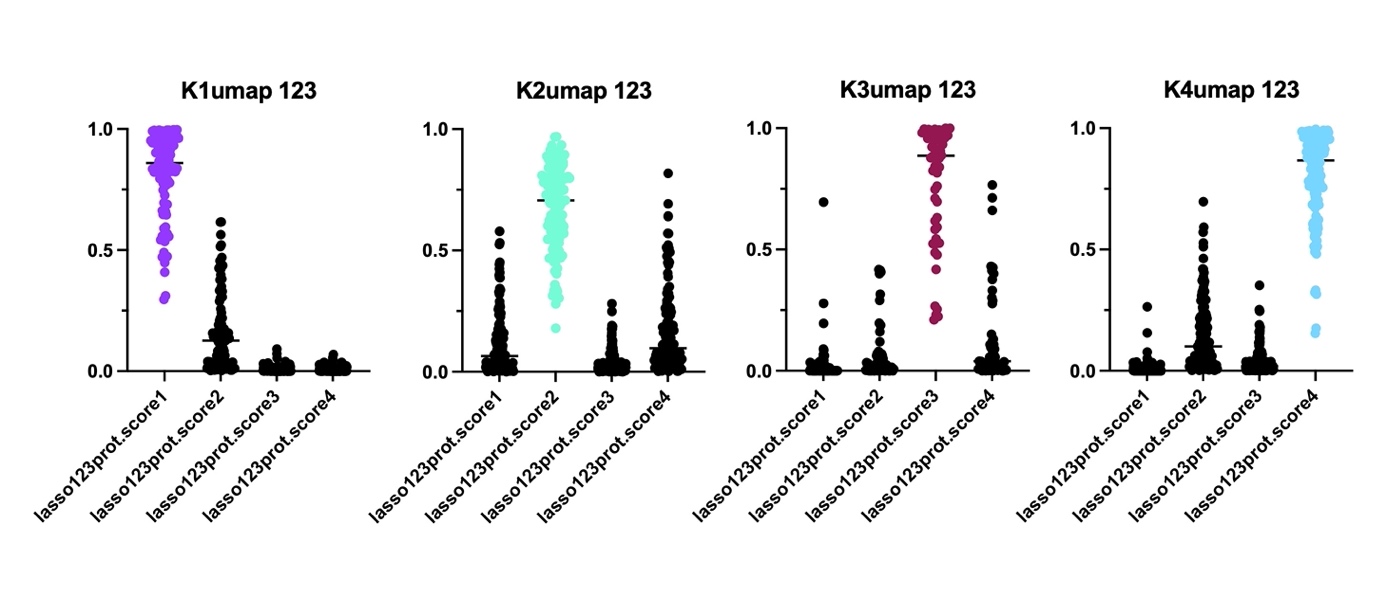
**

**Figure S13: Lasso scores according to cluster identified by random forest in UK (A) and French (B) validation cohorts.**

**
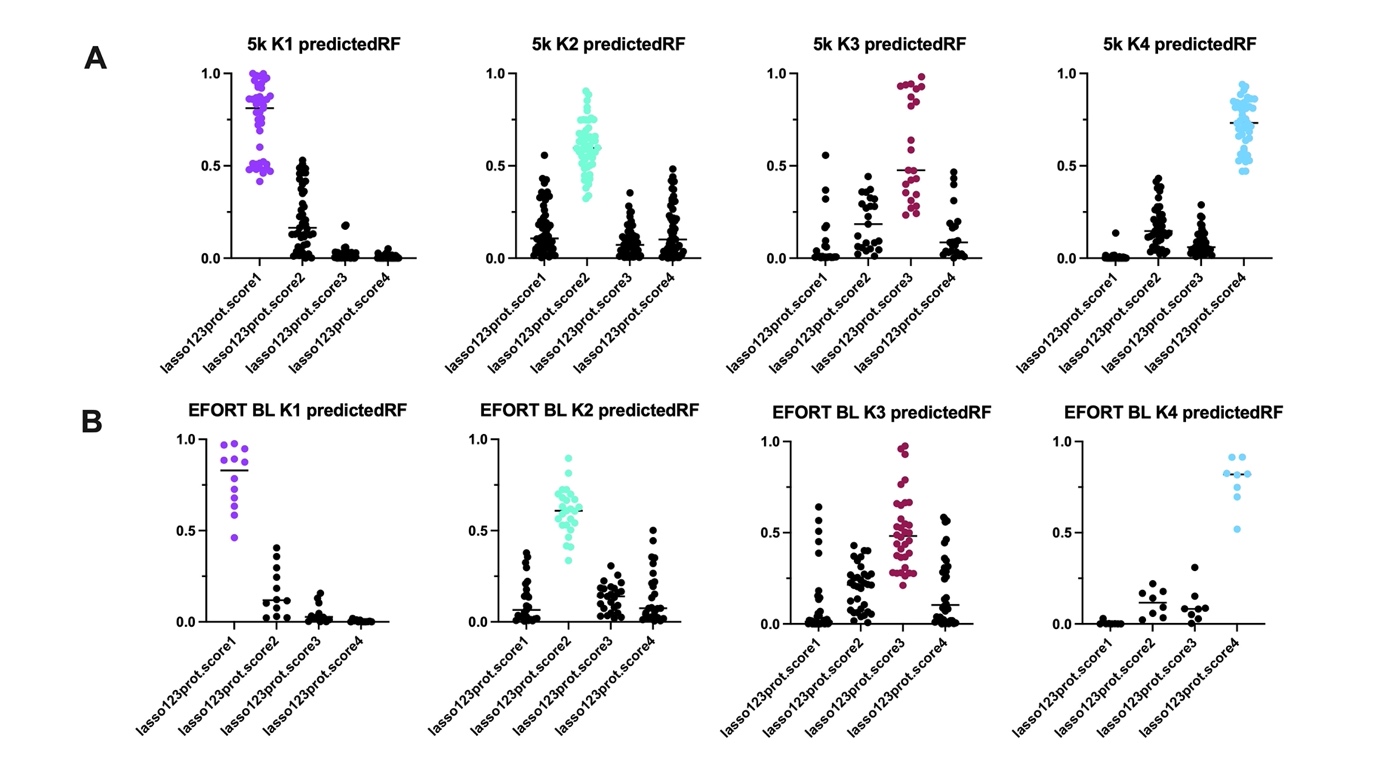
**

**Figure S14: Kaplan-Meier survival curves according to clusters in the UK validation cohort of patients with PH-LHD (A) and UK PH cohort with both precapillary and postcapillary PH (B)**

**
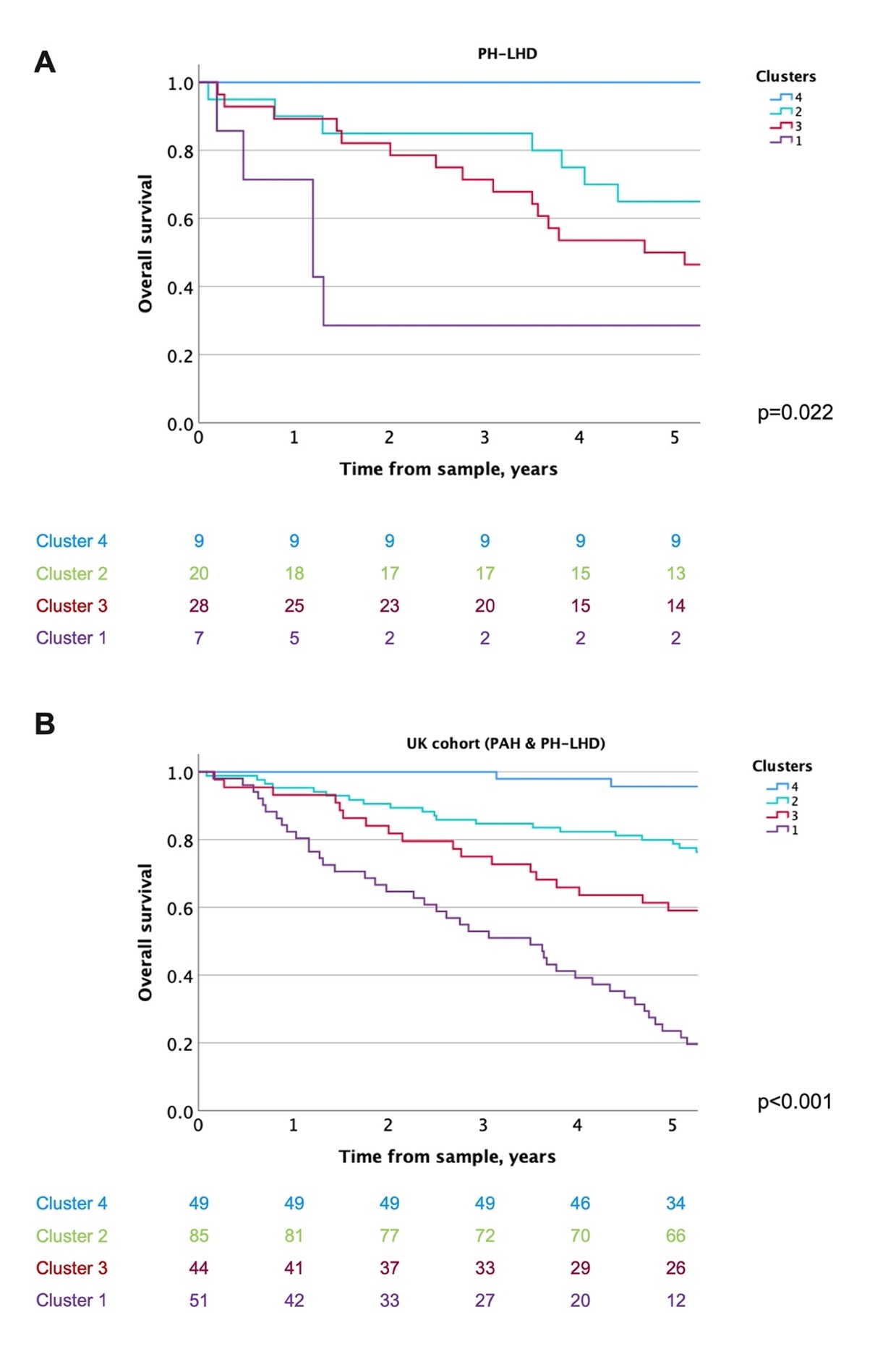
**

**Figure S15: Heatmap of somamers used to identify clusters in the Whitehall II cohort**

**
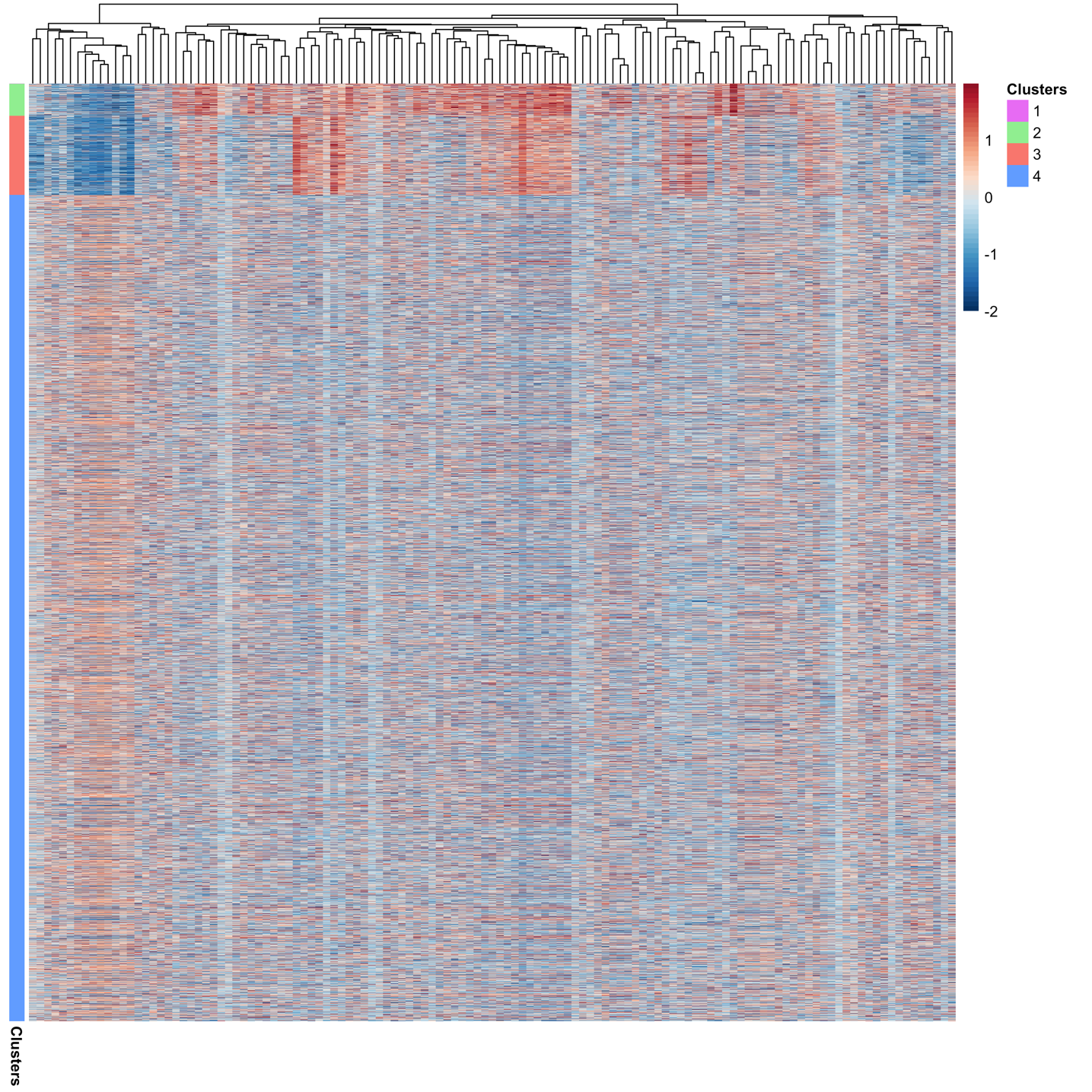
**
